# Nucleoside modifications in the regulation of gene expression: focus on tRNA

**DOI:** 10.1007/s00018-016-2217-y

**Published:** 2016-04-19

**Authors:** Markus Duechler, Grażyna Leszczyńska, Elzbieta Sochacka, Barbara Nawrot

**Affiliations:** 1 Centre of Molecular and Macromolecular Studies, Polish Academy of Sciences, Sienkiewicza 112, 90-363 Lodz, Poland; 2 Institute of Organic Chemistry, Lodz University of Technology, Zeromskiego 116, 90-924 Lodz, Poland

**Keywords:** Regulation of gene expression, Modified nucleosides, Stress signaling, tRNA, Translation rate

## Abstract

Both, DNA and RNA nucleoside modifications contribute to the complex multi-level regulation of gene expression. Modified bases in tRNAs modulate protein translation rates in a highly dynamic manner. Synonymous codons, which differ by the third nucleoside in the triplet but code for the same amino acid, may be utilized at different rates according to codon–anticodon affinity. Nucleoside modifications in the tRNA anticodon loop can favor the interaction with selected codons by stabilizing specific base pairs. Similarly, weakening of base pairing can discriminate against binding to near-cognate codons. mRNAs enriched in favored codons are translated in higher rates constituting a fine-tuning mechanism for protein synthesis. This so-called codon bias establishes a basic protein level, but sometimes it is necessary to further adjust the production rate of a particular protein to actual requirements, brought by, e.g., stages in circadian rhythms, cell cycle progression or exposure to stress. Such an adjustment is realized by the dynamic change of tRNA modifications resulting in the preferential translation of mRNAs coding for example for stress proteins to facilitate cell survival. Furthermore, tRNAs contribute in an entirely different way to another, less specific stress response consisting in modification-dependent tRNA cleavage that contributes to the general down-regulation of protein synthesis. In this review, we summarize control functions of nucleoside modifications in gene regulation with a focus on recent findings on protein synthesis control by tRNA base modifications.

## Introduction

Eukaryotic gene expression is controlled at many levels and modified nucleosides participate at several stages. DNA cytosine methylation regulates transcription rate through promoter CpG island methylation. Promoter methylation is lower in transcriptionally active genes. In addition, DNA cytosine methylation cooperates with histone modifications which determine the degree of DNA packaging into strings of nucleosomes and thus the general accessibility of the DNA for transcription [1]. The post-transcriptional regulation of gene expression affects the maturation, nuclear export and stability of messenger RNAs (mRNAs), and the control of protein translation initiation. mRNAs are synthesized as precursors (pre-mRNAs) which are the substrates for mRNA processing [2] including splicing [3], 5′-end capping, and 3′-end poly-adenylation. A methylated guanine nucleoside (m7G) linked to the mRNA through a 5′-5′ triphosphate linkage builds the 5′-cap which is required for the nuclear export. In the cytoplasm, the stability of mRNAs is regulated by the length of the polyA tail and by RNA interference (RNAi) mechanisms [4]. Furthermore, RNA base modifications such as *N*(6)-methyladenosine (m6A) influence the decoding process, as well as the stability and function of mRNAs through interaction with specific RNA binding proteins [5, 6]. The translation initiation is considered to be the rate-limiting step of peptide synthesis and is regulated by the availability of translation initiation factors and specific sequence elements within the 5′- and 3′-UTRs of the mRNAs [7]. tRNAs play a central role in the control of protein translation rates due to their function at the interface between mRNA and protein sequence, and tRNA nucleoside modifications contribute as important regulatory elements [8, 9]. In this review, we provide a short summary and an update about the influence of dynamic base modifications in various RNA species on protein translation with a focus on tRNA.

## The functional diversity of tRNAs

**Fig. 1:**
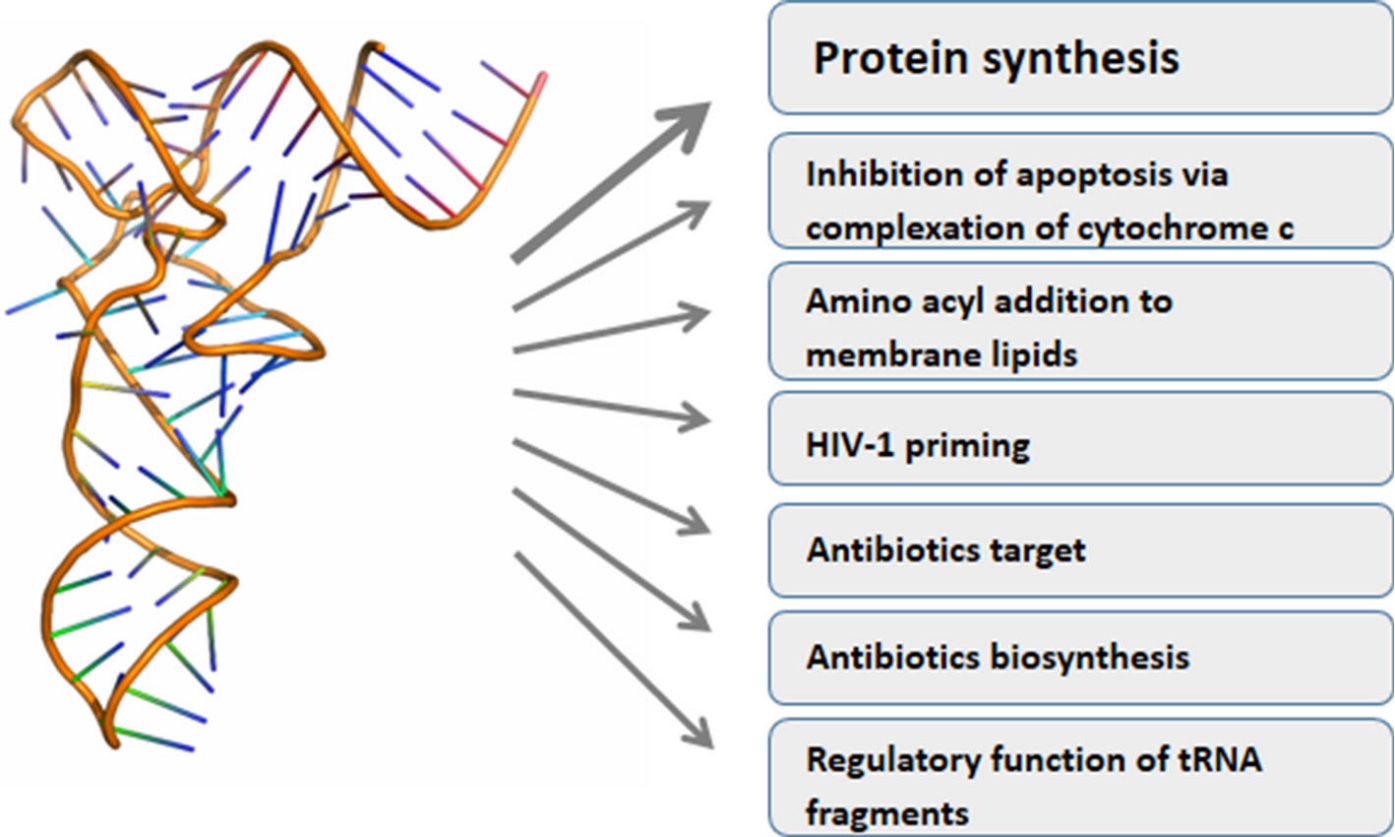
Functional diversity of tRNA (the tRNA structure was taken from RCSB protein data bank (PDB), file 3IOu)

Classically, tRNAs link mRNA and protein sequence information carrying amino acids to the ribosomes according to codon recognition. Research of the last years greatly increased the palette of tRNA functions (Fig. 1). For instance, tRNAs are used for adding amino acids to other substrates than the growing peptide chain, such as membrane lipids, proteins or antimicrobial agents [10]. Under stress some tRNAs are cleaved into halves creating molecules with signaling function [11], while shorter tRNA derived fragments can function as miRNAs [12–16]. Double stranded RNA fragments cleaved from the mature tRNAs repressed the translation of specific target mRNAs in mature human B cells [14], suppressed the expression of endogenous viruses [15, 16] or were required for proliferation of a colorectal carcinoma cell line [12].

**Fig. 2:**
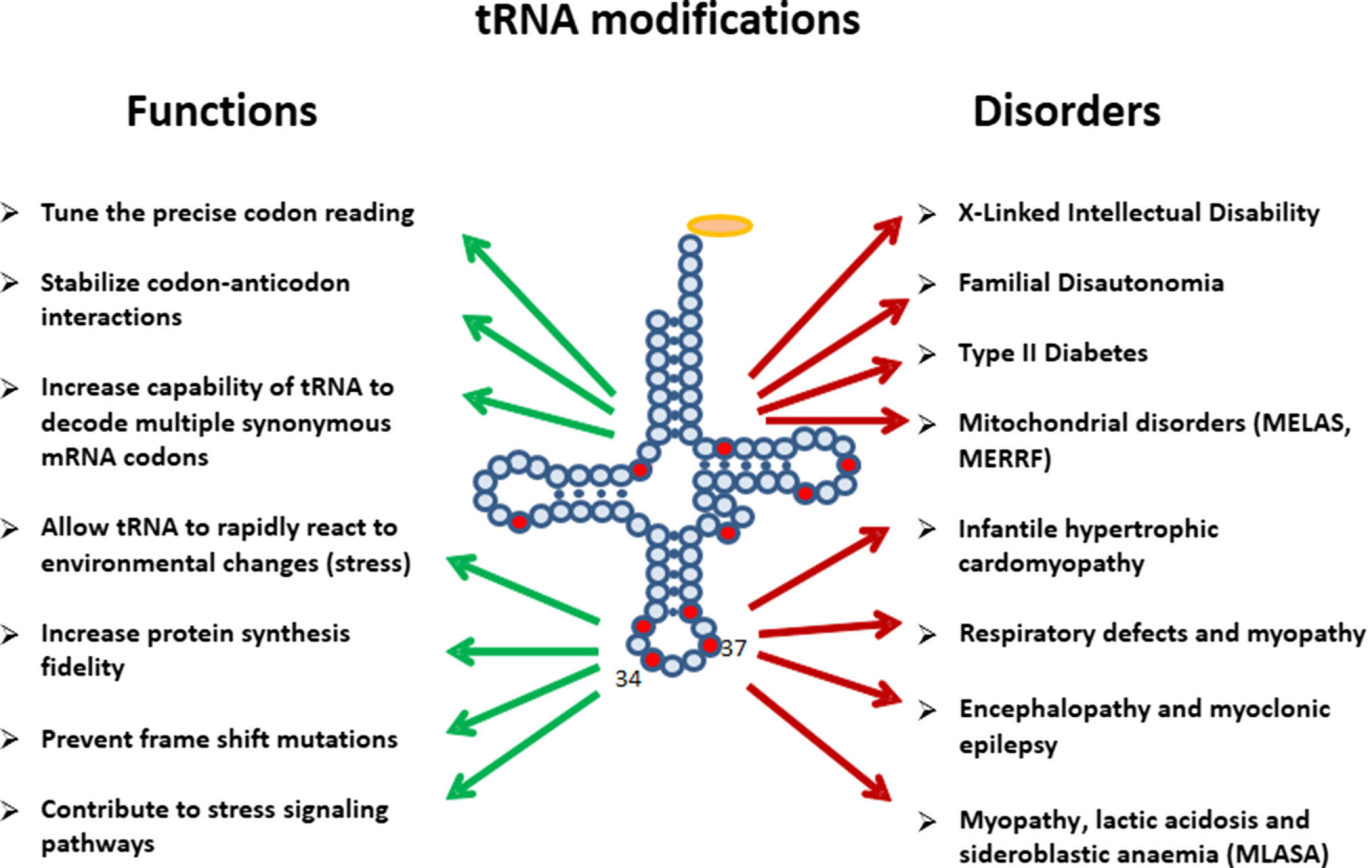
Functions of tRNA modifications

The tRNA molecules carry a majority of post-transcriptionally modified nucleosides, as they contain almost 90 out of total ca. 150 found in DNA and various classes of RNA (http://modomics.genesilico.pl; http://mods.rna.albany.edu) [17, 18]. Some tRNA base modifications are required to stabilize their tertiary structure. Consequently, they also influence aminoacylation rate and accuracy [19]. Other base modifications participate in the regulatory functions of tRNAs (Fig. 2). tRNA modifications were demonstrated to control tRNA cleavage [20–23]. Modifications influence the tRNA-mRNA interaction to either stabilize cognate Watson–Crick base pairing or to facilitate wobble base pairing to increase the coding capacity [24]. Base modification-mediated strengthening of codon–anticodon binding also prevents frame-shift errors [25]. Importantly, some base modifications show dynamic changes triggered by, e.g., stages in circadian rhythms, in cell cycle progression, or by environmental factors such as nutrition or stress [26].

### Various functions of tRNA derived fragments in stress responses

Stressed eukaryotic cells rapidly shut down the overall translation rate [27]. It was found that in certain stress conditions, tRNA halves are generated by cleavage of tRNA molecules in the anticodon loop [28]. In mammalian cells, the cleavage reaction is carried out by angiogenin, a secreted ribonuclease with angiogenic function (induction of blood vessel growth). In normal conditions, angiogenin is spatially segregated from cytoplasmic tRNAs and bound to its specific inhibitor, RNH1, which controls localization and activity of the enzyme [29]. Under oxidative stress, angiogenin is released into the cytoplasm. After activation, angiogenin first cleaves the 3′-CCA termini of tRNAs which prohibits the usage of tRNAs in translation [30]. This cleavage is reversible and becomes quickly repaired. Cleavage in the anticodon loop, however, creates tRNA halves and induces stress granule formation where those mRNAs not needed for the stress response are captured into an inactive state [31]. Also angiogenin becomes concentrated in stress granules [32]. Stress granule formation depends on the presence of 5′-tRNA halves, the 3′-halves being inactive in this regard. Stress granule formation can be connected to autophagy as has been clarified recently in yeast. In a process named granulophagy, stress granules are cleared in lysosomes [33].

In bacteria and yeast strains, the cleavage of the anticodon loop has a different function, it is used as defense mechanism to inhibit competing species. It is exerted by tRNA specific nucleases, including the bacterial toxins Colicin E5, Colicin D, and PrrC, as well as Zymocin and PaT from yeast [34–37]. The fungal anticodon nuclease PaOrf2 cuts the anticodon loop twice to prevent tRNA repair which is not possible after two sequential incisions [38].

Importantly, the presence or absence of tRNA modifications controls tRNA cleavage. In yeast cells for instance, the final methylation forming the 5-methylcarboxymethyl (mcm5) modification of uridine in a subset of tRNAs added by the tRNA-methyltransferase Trm9 transfers resistance to cleavage by PaT [20]. In the absence of cytosine-5 methylases, tRNA^Gly^ and tRNA^Asp^ derived 5′-halves cleaved from non-methylated tRNAs were selectively enriched in mouse and human [39]. In mammalian cells, lack of methylation caused by UV light exposure resulted in accumulation of tRNA halves processed by angiogenin and the cleavage could be inhibited by the cytosine-5 tRNA methyltransferase NSun2 [23]. tRNA cleavage by angiogenin could also be prohibited by cytosine-5 methylation exerted by DNA methyltransferase 2 (DNMT2) [21, 22]. The tRNA cleavage in response to oxidative stress was preceded by a conformation change which was demonstrated using an 1-methyladenosine (m1A) antibody specific for unfolded tRNA [40]. Tissue damage after ischemic reperfusion, toxic injury, and irradiation increased the concentration of tRNA halves in the human circulation. The tRNA fragmentation could be detected before DNA damage providing a diagnostically useful early indicator for organ damage [40].

The cleavage of tRNAs in stress conditions does not significantly reduce the levels of full-length tRNAs, only a small portion of tRNAs is split. Thus, rather than diminishing the availability of tRNAs for translation, tRNA halves exert signaling functions or inhibitory interactions with the translation machinery. Some tRNA fragments created by anticodon loop cleavage can bind to and inhibit proteins required for translation to decrease the global protein synthesis rate. An example are the fragments derived from two tRNA^Gly^ isoacceptors after cleavage by angiogenin. These 5′-tRNA halves contain a tract of at least four G-residues at the 5′-end and bind eIF4G, a component of the cap-binding eIF4F complex [41]. The importance of accurate tRNA cleavage control is further underlined by the fact that accumulation of 5′-tRNA halves is associated with neurological abnormalities in mice and humans as a consequence of neuronal loss [23].

The 5′-tRNA halves derived mainly from tRNA^Gly^ and tRNA^Val^ were found in the circulation of mice also in unstressed conditions [42]. Interestingly, their concentration was higher than that of serum miRNAs and changed with age suggesting a physiological role in the organism. This hypothesis gains further support by the fact that the corresponding 3′ halves were found only in trace quantities [43]. The predominance of 5′-tRNA halves over the 3′-halves was impressively confirmed in a study characterizing small RNA species in the circulation of cattle. Of more than 3 million tRNA-matched sequence reads, 72.8 % represented 5′-tRNA halves while the proportion of 3′-tRNA halves was less than 0.02 % [44]. Recently it was shown that the length and relative abundance of tRNA fragments in the human circulation is heterogeneous and depends on gender and race [45]. A direct influence of tRNA fragments on the mRNA stability of specific transcripts was demonstrated lately. Anticodon loop containing fragments derived from tRNA^Glu^, tRNA^Asp^, tRNA^Gly^, and tRNA^Tyr^ displaced the RNA-binding protein YBX1 from the 3′-UTR of several mRNAs resulting in their destabilization and abrogation of their function in breast cancer progression [43].

Besides the down-regulation of global protein synthesis, other fascinating functions of tRNAs and tRNA halves were described. Mitochondrial and cytoplasmatic tRNAs and tRNA halves were able to inhibit apoptosis through binding to cytochrome C inhibiting its pro-apoptotic signaling function [46, 47]. Furthermore, tRNA^Val^- and tRNA^Gly^-derived fragments were increased in ischemic rat brains and negatively regulated angiogenesis [48]. In breast and prostate cancer cells, sex hormone signaling enhanced tRNA cleavage by angiogenin and the produced fragments augmented proliferation [49].

There is a very interesting possible interconnection between the tRNA halves derived from slicing mature tRNA molecules and the evolution of tRNA which left its traces in the genomic organization of the tRNA genes: some tRNAs are encoded by split genes, many others contain an intron in the anticodon loop sequence so that the genes encoding the mature tRNAs are presented like divided into halves [50, 51].

## tRNA modifications in protein translation control

### Biased codon usage for translation fine-tuning

In general, the availability of tRNAs constitutes a primary control mechanism for the speed of protein synthesis. Furthermore, the codon–anticodon binding affinity, which can be modulated by tRNA base modifications, regulates the speed and fidelity of translation (Fig. 2) [24, 25]. Certainly, modifications in or around the anticodon loop of the tRNA molecule exert the strongest influence on translations rate and accuracy. The tRNA position 34, the wobble base complement, as well as position 37 adjacent to the anticodon triplet are very frequently modified to stabilize specific codon—anticodon interactions during the decoding process. Loss of this stabilizing effect due to deficiencies in base modifications can be pathologic and result in severe diseases such as nonsyndromic X-linked intellectual disability and familial dysautonomia [52–55]. Deregulation of RNA modification pathways has further been linked to type II diabetes and mitochondria-based pathologies (Fig. 2) [56].

tRNA nucleoside modifications in and around the anticodon loop are employed by the cell to selectively alter the spectrum of proteins which are preferentially synthesized. How does this work? The redundancy of the genetic code allows to choose from alternative codons for the same amino acid. Different mRNAs vary in the composition of their synonymous codons: for a given amino acid, codon usage is biased in many proteins towards a higher frequency of certain codons above others. The coordination of the concentration of a particularly modified tRNA with the frequency of its cognate codon in the mRNAs introduces an additional level of regulation to fine-tune translation and influences the translation rate for each protein [8, 57]. Furthermore, the 64 possible codon variants outnumber the amount of tRNA molecules raising the necessity of wobble base pairing [58]. Nucleoside modifications at the wobble position 34 of tRNA increase the capabilities of tRNA to decode multiple synonymous mRNA codons differing by the third nucleoside. tRNA modifications through modulating the codon–anticodon affinity regulate also the biased translation of mRNA subsets, and adjusting the frequency of tRNA modifications allows the cell to rapidly react to environmental changes including different kinds of stress, utilizing the protein synthesis capacity for the actually most needed proteins. In *E. coli*, stress resistance was altered when synonymous mutations were introduced into heat shock genes [59].

The elaboration of the interconnection between tRNA nucleoside modification and translation fidelity followed different but complementing experimental paths: (1) observation of particular phenotypes including specific diseases triggered the discovery of the underlying defects of enzymes responsible for nucleoside modifications in tRNAs; (2) experimental deletion of genes encoding for nucleoside modifying enzymes induced particular cellular dysregulations; (3) hypersensitivity to various stress conditions were connected to hypomodified tRNAs. In the following section, we would like to highlight some recent findings connecting tRNA modification with translation features.

### Codon–anticodon interactions are affected by tRNA modified nucleosides to regulate cytoplasmic protein translation

Already in 2007, modifications of tRNA wobble nucleosides have been linked to the preferential translation of some transcripts with particular arginine and glutamic acid codons [60]. Trm9, a yeast methyltransferase, completes the synthesis of 5-methylcarboxymethyl-2-thiouridine (mcm5S2U) by adding the final methyl group. The affected uridines were found in the wobble position of tRNA^Arg^ (UCU) and tRNA^Glu^ (UUC), and Trm9 activity enhanced the synthesis of DNA damage response proteins [60]. Since then, evidence continuously accumulated that the content of overall tRNA nucleoside modifications was associated with translation efficiency for particular proteins [61]. During the recent years, it was shown that lack of URM1- and ELP-dependent tRNA wobble modifications decreased the translation of mRNAs enriched in Lys-AAA, Gln-CAA and Glu-GAA codons. Both, thiolation and methoxycarbonylmethylation of uridines in position 34 enhanced the binding of tRNAs to the ribosome and in this way promoted translation of mRNAs rich in the favored codons [62]. Modifications in tRNA anticodon loops are necessary to prevent frame-shift mutations. In eukaryotes, the wobble uridine modifications mcm5 and S2 side groups at wobble uridines are important for reading frame maintenance [63]. The phenotypes arising when U34 lacks sulfur at position 2 or the side chain at position 5 can be suppressed by overexpression of hypomodified tRNAs. Metabolic deficiencies in fission yeast were caused by changes in mRNA decoding due to the lack of isopentenyl-A (i6A) modification in position 37 [64].

A recent example of a newly discovered tRNA modification frequently found at position 37 is a cyclic form of *N*6-threonylcarbamoyladenosine (ct6A) [65]. The modification is required for correct decoding of ANN codons. *N*6-threonylcarbamoyladenosine (t6A), a modification well-known since several decades, might have been an artifact resulting from hydrolysis of the native ct6A in the conditions used for nucleoside isolation. In physiological conditions, ct6A shows remarkable stability [66].

### Importance of wobble uridines/2-thiouridines in codon–anticodon interaction

**Fig. 3:**
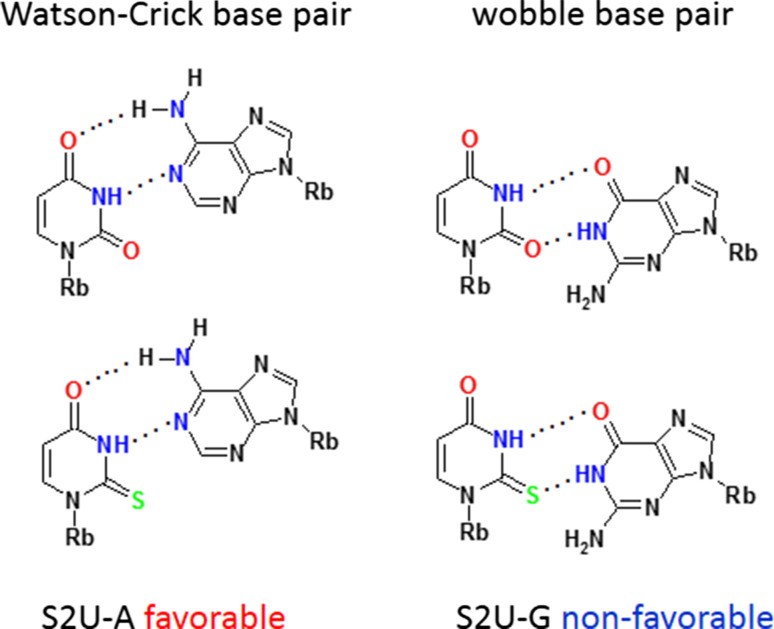
The Watson–Crick and wobble base pairs of uridine and 2-thiouridine with adenosine and guanosine

About 40 different modified uridines and 2-thiouridines are found in the first (wobble) position of the anticodon. According to the original Crick’s hypothesis uridine can form a base pair with either adenosine or guanosine (Fig. 3) [67]. Replacement of oxygen-2 of the uracil ring with a sulfur atom is observed for at least ten wobble uridines in tRNAs specific for lysine, glutamic acid and glutamine. Many uridines at the wobble position are substituted with a side chain at the carbon C5. Both, the presence of sulfur and the modifications at C5 control the decoding behavior [11]. 2-Thiouridines, besides their significant contribution to translational regulation through codon–anticodon interactions, serve as recognition elements for cognate aminoacyl-tRNA synthetases (aa-RS) [19, 68] and are used together with other modifications by the innate immune system to distinguish between self and pathogen RNAs [69–71]. Furthermore, the thiocarbonyl function of S2U-containing human tRNALys3 is involved in the formation of the initial complex with HIV-1 RNA as a prerequisite for viral RNA reverse transcription [72]. Notably, 5-methyl-2-thiouridine (or 2-thioribothymidine) is also located in the TΨC loop of tRNAs of hyperthermophilic species such as *Thermus thermophilus* [73, 74] and *Pyrococcus furiosus* [75]. This nucleoside is thought to enhance the stability of the tertiary structure of tRNA at elevated temperatures.

The thiolation status of wobble uridines directly depends on the intracellular methionine and cysteine availability [76]. Furthermore, a kind of intra-molecular cross-regulation was discovered in yeast. A temperature-sensitive mutant showed reduced levels of 2-thiolated U34 in tRNA^Gln^ (UUG) as a consequence of the lacking pseudouridine (Ψ) 38 modification [77]. The 2-thiouridine (S2U) units facilitate Watson–Crick base pairing with adenosine and the wobble pairing with G in the third position of the codon and restrict reading of near-cognate codons [58, 78]. Affinity measurements showed that the S2U modification of tRNA^Gln^ significantly strengthens the binding to glutamine codons compared to unmodified uridine to enhance aminoacylation kinetics and to prevent frame-shift errors [63, 79]. We reported previously that S2U nucleosides can be dethiolated in the presence of hydrogen peroxide resulting in a mixture of uridine and the 4-pyrimidinone riboside (H2U) [79]. Further analysis revealed that the product ratio depends on the pH of the reaction mixture [80] and on the substituent at position 5 of the nucleobase [81]. When S2U is transformed into H2U, the preferred base pairing with adenosine is lost in favor of base pairing with guanosine [82]. Recently we have proved that desulfuration process in S2U-RNA in the presence of hydrogen peroxide is catalyzed by cytochrome c (unpublished results). Moreover, experiments carried out on yeast cultures treated with hydrogen peroxide have shown that about 7 % of mcm5S2U-tRNA is desulfurated, mainly into the prevailing mcm5H2U-tRNA product and only into a minor amount of mcm5U-tRNA (unpublished).

### Methyltransferases

Many tRNA base modifications consist of single methylations, or—in case of more complicated structures—include a methylation step. Therefore, tRNA methyltransferases are a class of intensively investigated enzymes. Methylation of guanosine in tRNA position 37 carried out by TrmD was found in many species and acts as frameshift suppressor [83]. In higher eukaryotes, DnmT2 and NSun2 transfer methyl groups to the cytosine 5 position [39]. Knock-out of both enzymes resulted in a complete loss of m5C, reduced protein synthesis rates and was lethal for mice [84] and yeast cells [85]. Cytosine methylation in tRNA^Asp^ position 38 is important for correct amino acid incorporation to properly discriminate between Asp and Glu codons [84]. Other methyl transferases are Abp140 in yeast and NSun6 in humans, producing 3-methylcytidine (m3C) found in tRNA position 32 and m5C in position 72, respectively [86, 87]. Methylations constitute also a major class of modifications of mitochondrial tRNAs.

### Mitochondrial tRNA modifications

Mitochondria are semi-autonomous organelles that possess their own genomes as well as transcription and translation machinery to provide the chemical energy required by living cells. The human mitochondrial DNA (mtDNA) encodes 13 proteins of the oxidative phosphorylation system (OXPHOS), two rRNAs, and all of the 22 tRNAs (mt-tRNA) required for mitochondrial protein synthesis. This small (16.5 kb) mt-DNA has multiple copies in mitochondria; however, it is not able to express all the components needed to sustain a functional translation machinery. The remaining RNAs and proteins are encoded in the nucleus, expressed in the cytosol and then imported into mitochondria, including proteins required for synthesis and regulation of mt-DNA, mitochondrial ribosomal proteins, aminoacyl-tRNA synthetases and tRNA-modifying enzymes [88–92].

To became functional elements of mitochondrial protein biosynthesis, mt-tRNAs undergo numerous post-transcriptional modifications [93–96]. Modifications act directly on the structure, stability and consequently the functionality of mt-tRNA [52, 97–99]. Modified nucleosides play key roles in the folding process of mt-tRNAs to provide the functional tertiary L-shaped structure [97]. The modifications involved in this process are commonly base-methylated purines, e.g., m1A, m1G, and m2,2G. The methyl group attached to bases prevents formation of false Watson–Crick pairings and wrong folding of mt-tRNAs. Usually, several post-transcriptional modification steps are needed to ensure the correct mt-tRNA folding. Surprisingly, the presence of 1-methyladenosine (m1A) at position 9 is a necessary and sufficient requirement for the correct folding of human mt-tRNA^Lys^ to the canonical tertiary structure by prohibiting the pairing between A9 and U64 [97, 100, 101].

**Fig. 4:**
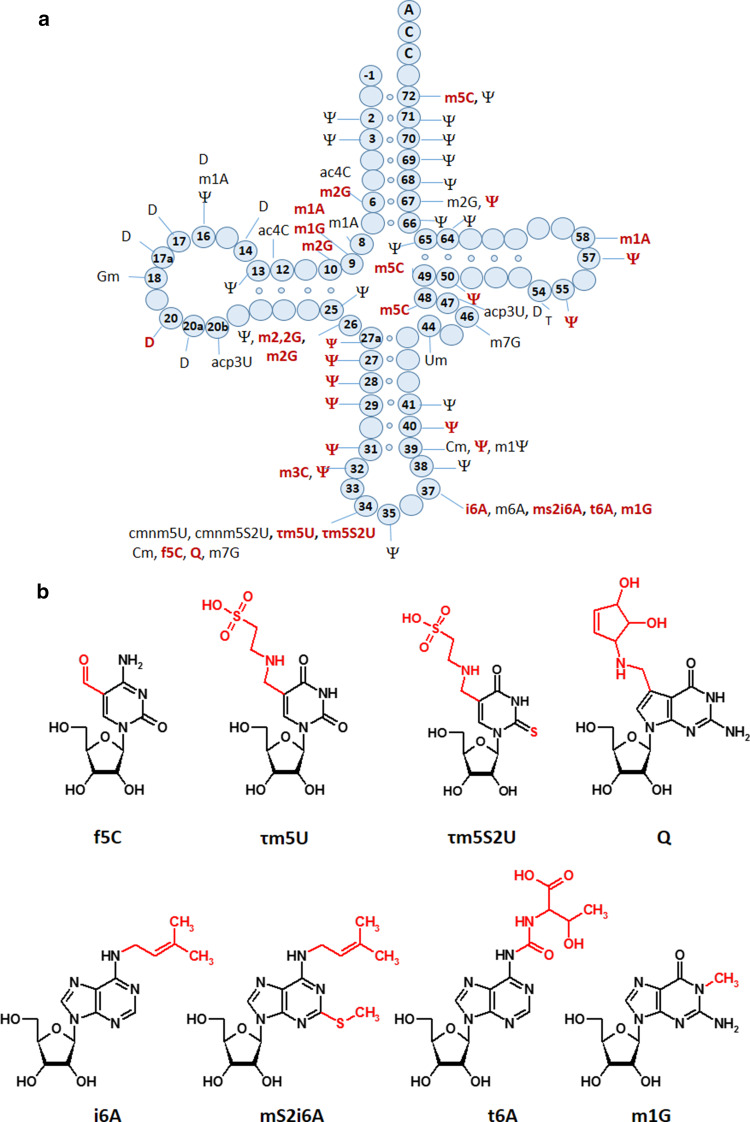
Mitochondrial tRNA nucleoside modifications. **a** Distribution of post-transcriptional modifications in mt-tRNA [90, 95]; modifications identified in mammalian mt-tRNAs are indicated in *red* [94]; **b** chemical structures of mitochondria-specific modifications located at the anticodon wobble position: 5-formylcytidine (f5C), 5-taurinomethyluridine (τm5U), and 5-taurinomethyl-2-thiouridine (τm5s2U); and in position 37: *N*6-isopentenyladenosine (i6A), 2-methylthio-*N*6-isopentenyladenosine (ms2i6A), *N*6-threonylcarbamoyladenosine (t6A), and 1-methylguanosine (m1G)

tRNA modifications are essential for the decoding capacity, recognition by aminoacyl-tRNA synthetases, translation factors and mt-tRNA modifying enzymes [92, 93, 95, 102–104]. In mammals, 16 different modifications were identified in 23 positions of all 22 mt-tRNAs (Fig. 4); however, the greatest diversity of modifications was found in nucleosides located at position 34 and 37. The mammalian mitochondrial decoding system differs from the universal genetic code. Mitochondria use four non-universal codons: AUA for Met instead of Ile, UGA for Trp instead of STOP and AGR (R = A or G) for Stop instead of Arg [93, 94]. The reduced number of mt-tRNAs in comparison with the cytosolic system of translation requires a more flexible codon reading. Thus, post-transcriptional modifications located at the wobble position of mt-tRNAs play a critical role in efficiency and accuracy of this minimal decoding system [105]. Recently, Suzuki et al. demonstrated a first landscape of modifications in all 22 species of mt-tRNAs isolated from bovine liver [95]. Codon–anticodon pairing analysis revealed that eight mt-tRNAs bearing Leu, Val, Ser, Pro, Thr, Ala, Arg and Gly were found to read family box codons. In all these cases, the conformational flexibility of unmodified U located at position 34 enables reading of all four bases at the third codon position. The remaining mt-tRNAs are responsible for recognition of two codons each, including the purine-ending codons NNA and NNG. Their post-transcriptional wobble modifications refer to mitochondria-specific 5-substituted pyrimidines: 5-taurinomethyluridine (τm5U), 5-taurinomethyl-2-thiouridine (τm5S2U) and 5-formylcytidine (f5C) identified in mt-tRNA^(Leu, Trp)^, mt-tRNA^(Lys, Gln, Glu)^ and tRNA^Met^, respectively [104, 105, 106]. It has been proposed that uridine derivatives of the m5(S2) type restrict the conformational flexibility of the wobble uridine preventing misrecognition of the near-cognate codons ending in cytidine or uridine [93]. Defects in the taurine modified uridines of human tRNA^Leu^(τm5UAA) and mt-tRNA^Lys^(τm5S2UUU) are associated with two major classes of mitochondrial diseases, MELAS and MERRF caused by point mutations in mt-RNA genes (Fig. 2) [52, 93, 107, 108–112]. The pathogenic mutations located in the D or T loop of tRNA^Leu^ and mt-tRNA^Lys^, respectively [93, 105, 113–115] are supposed to disrupt the tertiary structure of mt-tRNAs that prevents their recognition by enzymes responsible for τm5(S2)U biosynthesis [116, 117]. The taurine-containing modifications are believed to play an important role in stabilizing codon–anticodon interactions in the pairing with A/G as the third codon letter [118]. The absence of τm5U results in a defect of translating UUG-rich genes. Among them is ND6 (NADH-ubiquinone oxidoreductase chain 6) located in Complex I of the respiratory chain. The τm5S2U deficiency results in the complete loss of pairing with both cognate codons AAA and AAG, and reduction in Complex I and IV activities. According to previous data [85, 119–121], the presence of a 2-thiocarbonyl group and a taurinomethyl residue in the structure of wobble τm5S2U seems to be crucial for optimal mammalian mitochondrial translation.

Very similar codon–specific translational defects were also observed in the absence of 5-carboxymethylaminomethyl-2-thiouridine (cmnm5S2U) located at the wobble position of *S. cerevisiae* mt-tRNA bearing Lys, Glu and Gln [122]. The lack of the cmnm5U_34_ and 2-thiocarbonyl modifications caused the complete degradation of mitochondrial tRNA, mRNA, and rRNA, instability of mitochondrial genome, perturbation in mitochondrial translation and consequently, respiratory defects.

Recent reports suggest that incorrect maturation of mt-tRNA^(Lys, Glu, Gln)^ resulting in the loss of taurine modified uridine and 2-thiouridine can be also caused by mutations in the genes for the mitochondrial translation optimization factor 1 (MTO1) and tRNA 5-methylaminomethyl-2-thiouridylate methyltransferase (TRMU) responsible for 5-methylaminomethylation and the thiolation of U_34_, respectively [90, 93]. Deficiency of the MTO1 enzyme was reported in patients with infantile hypertrophic cardiomyopathy whereas the mutated TRMU caused reversible hepatopathy [90, 123, 124]. The MTO1 mediated mt-tRNA modification has been recently presented as a novel factor in tissue-specific regulation of OXPHOS, fine-tuning of mitochondrial translation accuracy and balancing of mitochondrial and cellular secondary stress responses [125–127].

The ability of base modifications to regulate the codon–anticodon interactions and mt-tRNA biochemical functions was clearly exemplified by 5-formylcytidine (f5C) [107]. The modified cytidine located at the wobble position in mammalian mt-tRNA^Met^(f5CAU) is able to recognize the non-universal AUA and the canonical AUG codons. Both purine ending codons are decoded by a single tRNA^Met^ that is functional in the mitochondrial protein initiation and elongation steps [128, 129]. In the cytosol, a single AUG encoding methionine is recognized by two different tRNAs attending the initiation or elongation step. Thus, the presence of wobble f5C was suggested to expand the codon recognition capabilities of tRNA^Met^ through enhanced binding to the AUA codon [129, 130].

Contrary to wobble 5-substituted pyrimidines decoding the purine-ending codons, four mammalian mt-tRNA^(Tyr, His, Asn, Asp)^ modified with queuosine (Q) are responsible for recognition of pyrimidine-ending codons (NAU/NAC). Q_34_ is known to restrict the conformational flexibility of the anticodon loop by stabilization of the U-turn [131] and has been implicated in regulating the decoding capabilities of tRNA [132–134].

The frequency of modified nucleosides at position 37 (adjacent to the anticodon) is almost two times higher than at the wobble position [96]. Among all known purines identified at this position the most common are *N*6-isopentenyladenosine (i6A), *N*6-isopenetyl-2-thiomethyladenosine (mS2i6A), *N*6-threonylcarbamoyladenosine (t6A) and 1-methylguanosine (m1G). On the basis of the functional role of cytoplasmic tRNA modifications [52, 135–139], mitochondrial modified A_37_ are supposed to affect the behavior of tRNAs during translation via base-stacking stabilization of codon–anticodon interactions, prevention of translational frame-shifting and maintaining decoding accuracy. In fact, the 6-isopentenyl group of i6A_37_ was found to improve the efficiency and accuracy of mitochondrial translation [64]. The loss of i6A_37_ in human mitochondrial tRNAs has been recently described as a result of pathogenic mutations in the tRNA isopentenyl transferase (TRIT1), responsible for i6A_37_ biosynthesis. Defective i6A_37_ was reported in patients with encephalopathy and myoclonic epilepsy [140]. In some mammalian mt-tRNA sequences i6A_37_ is modified by a mitochondrially localized methylthiotransferase, CDK5RAP1, to form mS2i6A_37_ [141]. In cdk5rap1 knockout mice deficient in the 2-methylthio modification in mt-tRNAs bearing Ser(UCN), Phe, Tyr and Trp, impaired mitochondrial protein synthesis was observed causing respiratory defects of mitochondria and myopathy in vivo [142]. Furthermore, Wei et al. demonstrated that the 2-methylthio group in mS2i6A_37_ is sensitive to oxidative stress and was reduced in patients with mitochondrial disease [142].

The most abundant modification in mt-tRNA sequences is pseudouridine (Ψ), found at 27 positions of mt-tRNAs (Fig. 4) [96]. Due to its unique ability to coordinate a structural water molecule via the free N1-H, pseudouridine exhibits improved base stacking and thus, stabilizes higher order RNA structures by rigidifying the nearby sugar-phosphate backbone [143, 144]. Therefore, it is not surprising that Ψ is frequently found in characteristic structural motifs in tRNA and rRNA, such as the junctions of single-stranded and helical regions [145]. Ψ is the product of the non-reversible isomerization of uridine in the process called pseudouridylation. The U → Ψ conversion takes place within the RNA chain and is catalyzed by a family of pseudouridine synthases (PUS) as the “writers” of Ψ which employ both sequence and structural information to achieve site specificity [146]. Mutations in the PUS1 gene preventing the Ψ biosynthesis in human mitochondria cause myopathy, lactic acidosis and sideroblastic anemia (MLASA) [147, 148].

## tRNA modification dynamics

Some tRNA nucleoside modifications show dynamic changes (Table 1) caused by environmental factors such as stress or nutrition. During the stress response, a widespread reprogramming of tRNA modifications occurs [149]. The tRNA base modifications change in a characteristic manner depending on the kind of stress creating reproducible modification patterns which can even be used to distinguish among chemically similar stressors and to predict toxicant chemistry [150]. In a seminal publication, Chan et al. showed the dynamic changes of tRNA modifications during various cellular stress conditions in yeast [151]. They demonstrated that tRNA modifications such as Cm, m5C and m2,2G (*N*2,*N*2-dimethylguanosine) enabled cells to better survive in the presence of H_2_O_2_ and that the oxidative stress controls the degree of tRNA modifications. An increase in cytosine methylation upon oxidative stress was also shown for the yeast tRNA^Leu^ (CAA) which contains m5C at the wobble position, methylated by the Trm4 methyltransferase. The increased proportion of m5C containing tRNAs upon exposure to hydrogen peroxide resulted in increased translation of mRNAs enriched in the corresponding UUG codons [152]. Also in yeast tRNA^His^, the level of m5C was raised in different stress conditions and was accompanied by growth arrest [153]. In mammalian systems, oxidative stress caused an increase in the 5-methylcarboxymethyl-2′-*O*-methyluridine (mcm5Um) modification to promote the expression of selenocysteine containing proteins which contribute to detoxification of reactive oxygen species [154].

**Table 1:** Dynamic changes in tRNA base modifications are coordinated with cellular physiology

Nucleoside modification	Dynamic change	Effects
	Increased general levels in tRNAs after exposure of yeast cells to H_2_O_2_	Better survival in the presence of H_2_O_2_
	Increased levels at the wobble position of tRNA^Leu^ (CAA) and tRNA^His^ after exposure of yeast cells to H_2_O_2_	Increased translation of mRNAs enriched in the corresponding UUG codons Growth arrest in yeast
	Increased general levels in tRNAs after exposure of yeast cells to H_2_O_2_	Better survival in the presence of H_2_O_2_
	Increased levels in mammalian systems under oxidative stress	Promotion of the expression of selenocysteine containing proteins which contribute to detoxification of reactive oxygen species
	In the presence of H_2_O_2_, dethiolation occurred in vitro and in yeast cells	Change in decoding preferences? ‘Chemical’ cleavage of tRNA into tRNA halves?
	Oscillating levels during the cell cycle were observed	Decreased translation fidelity and activation of stress response pathways Cell division regulation Telomeric gene silencing and DNA damage response
	Levels change in a growth phase-dependent manner	High levels facilitate wobble base pairing with guanosine and pyrimidines

The levels of modified nucleosides change in oxidative stress conditions, with the cell cycle progression, or during different growth phases of bacteria

*Cm* 2'-*O*-methylcytidine, *m5C* 5-methylcytidine, *m2,2G* *N*2,*N*2-dimethylguanosine, *mcm5Um* 5-methylcarboxymethyl-2′-*O*-methyluridine, *S2U* 2-thiouridine, *H2U* pyrimidinone nucleoside, *cmo5U* 5-carboxymethoxyuridine, *mcmo5U* 5-methylcarboxymethoxyuridine

Lack of specific modifications can cause stress-sensitive phenotypes. The histone acetyltransferase Sin3/Elp3, a component of the Elongator complex, is required for the modification of the wobble nucleoside in tRNA^Lys^ (UUU). In fission yeast cells lacking Sin3/Elp3, the wobble U nucleoside in tRNA^Lys^, tRNA^Glu^ and tRNA^Gln^ is not modified which causes a phenotype highly sensitive to oxidative stress [155]. The anticodon loop modifications carried out by Sin3/Elp3 optimize translation efficiency for survival of oxidative stress. The dynamics can be further amplified by negatively affecting the translation of transcription factors such as Atf1 and Pcr1 which are required for the transcription of stress response genes [155]. The mRNAs for Atf1 and Pcr1 contain an elevated AAA:AAG ratio for Lys codons, thus they are more efficiently translated during stress than, e.g., house-keeping genes which in general show low AAA:AAG ratios [56]. In special situations, the lack of modifications can also be of advantage. It was demonstrated that defects in tRNA uridine thiolation confers resistance to ER stress due to a generally decreased translation rate [156].

### Influence of sulfur at C2 and C5-substitutions on the base pairing of wobble uridines/2-thiouridines

RNA duplexes containing a S2U-A base pair are thermodynamically more stable than those with a U-A base pair due to a preferential S2U C3′-*endo* sugar ring pucker, improved base stacking in RNA chains [157] and an enhanced overall A-type RNA duplex helical structure [158–166]. Early data [159] suggested that Nature had introduced 2-thiouridine into the wobble position to preserve hybridization to adenosine, and to restrict the wobble pairing with guanosine [167]: the wobble S2U-G base pair is less stable than U-G and the presence of sulfur favors S2U-A over S2U-G base pairs (Fig. 3).

However, the presence of C5 side chains can change the preference for U-A base pairing and biological studies suggested favored reading of the 3′-G-ending codons by anticodons when the 2-thiouridines contained 5-substitutions [168]. The substituent at the position C5, through its electron withdrawing/donating properties, contributes to the electron density within the π system of a nucleobase. This feature strongly depends on the geometry of the 5-substituent, especially if the substituent may interact with the nucleobase electron system not only through a mesomeric but also through an inductive effect [169].

Consequently, the loss of C5 modifications results in distinct phenotypes in yeast and bacterial cells. Deficiency of Trm9, a yeast methyltransferase, required for the synthesis of mcm5U and mcm5S2U, resulted in decreased translation fidelity and activation of stress response pathways caused by protein errors [170]. Yeast cells lacking both, the S2- and the mcm5-uridine modification at the wobble position showed reduced protein levels and growth impairment mainly due to tRNA^Lys^ hypomodification [171]. tRNA modifications also show regular changes in normal cellular processes, as their levels oscillate together with the cell cycle [172]. The Trm9-mediated methylation of mcm5S2U was higher during the S phase than in G1 and G2. The increase in the mcm5U modification during the S phase promoted cell cycle progression by upregulating the translation of the ribonucleotide reductase (RNR) complex which catalyses the conversion of ribo- into deoxyribonucleotides [172]. Consistent with these findings, Elongator was shown to be involved in cell division regulation [173]. Elongator is a protein complex consisting of six subunits conserved in eukaryotes, it has tRNA modification activity and seems to act upstream of Trm9 [174]. Elongator participates in the synthesis of mcm5 and 5-carbamoylmethyl (ncm5) modifications of uridines. In addition to its role in the cell cycle, the tRNA modification activity of the Elongator complex controlled the expression of proteins involved in telomeric gene silencing and DNA damage response [175].

In gram-negative bacteria, 5-carboxymethoxyuridine (cmo5U) and its methyl ester derivative 5-methylcarboxymethoxyuridine (mcmo5U) were found at the wobble position in several tRNAs [176]. Their level in tRNA^Pro3^ changed in a growth phase dependent manner. Both, cmo5U and mcmo5U were suggested to facilitate wobble base pairing with guanosine.

**Fig. 5:**
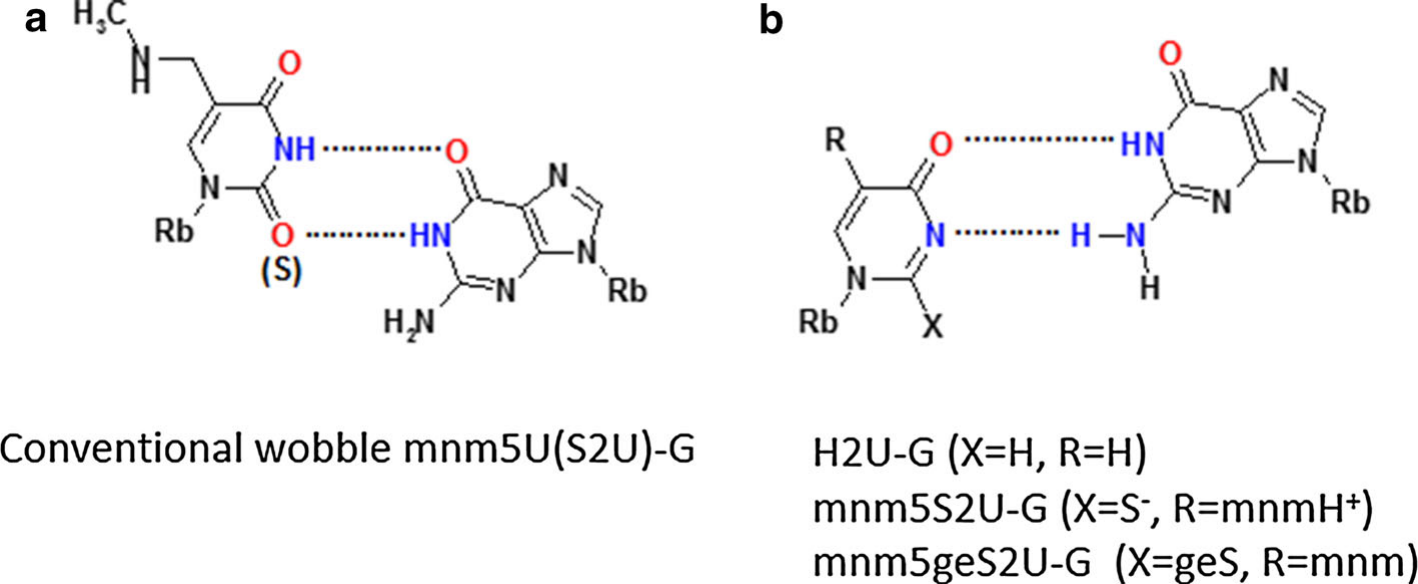
Base pairing between modified uridines and guanosine. **a** The conventional wobble base pair between mnm5U or mnm5S2U and guanosine; **b** base pairing of a H2U-type nucleoside with guanosine (X = H for H2U, X = S^−^ for a model proposed for a novel base pair between zwitter ionic mnm5S2U and guanosine, X = geS for mnm5geS2U)

Oxidative stress can result in loss of the sulfur atom of 2-thiouridine. In our studies on the oxidative desulfuration we obtained the 4-pyrimidinone products at the nucleoside (H2U, Fig. 5b, X = H) and RNA oligonucleotide (H2U-RNA) level [79]. Measurement of the binding affinity of H2U to A and G complements [79, 82] indicated that the H2U-G base pair is as stable as a U-G wobble base pair. This is only possible if it forms a base pair in the RNA duplex with two hydrogen bonds, which makes a movement of pyrimidine residue towards the minor groove necessary, as predicted by Takai [177]. These assumptions were confirmed very recently by the team of Yusupova [178], by crystallization of *Escherichia coli* tRNA_UUU_^Lys^ complexed with the 70S ribosome and with a short mRNA fragment. In this mnm5S2U-G base pair the thiouridine is prestructured into a zwitter ionic form as shown in Fig. 5b (with X = S^−^ and R = mnmH^+^/–CH_2_N^+^H_2_CH_3_). We also realized that the H2U model has the identical donor/acceptor pattern as *S*-geranyl-2-thiouridine [179] (Fig. 5b, X-geS). The geS2U nucleoside is a product of geranylation of a small fraction (ca. 7 %) of the wobble mnm5S2U and cmnm5S2U present in tRNA^Lys^, tRNA^Glu^ and tRNA^Gln^. The binding mode of geS2U with G is the same as in H2U-G, as suggested by us and others [11, 180].

## Base modifications of mRNA

**Fig. 6:**
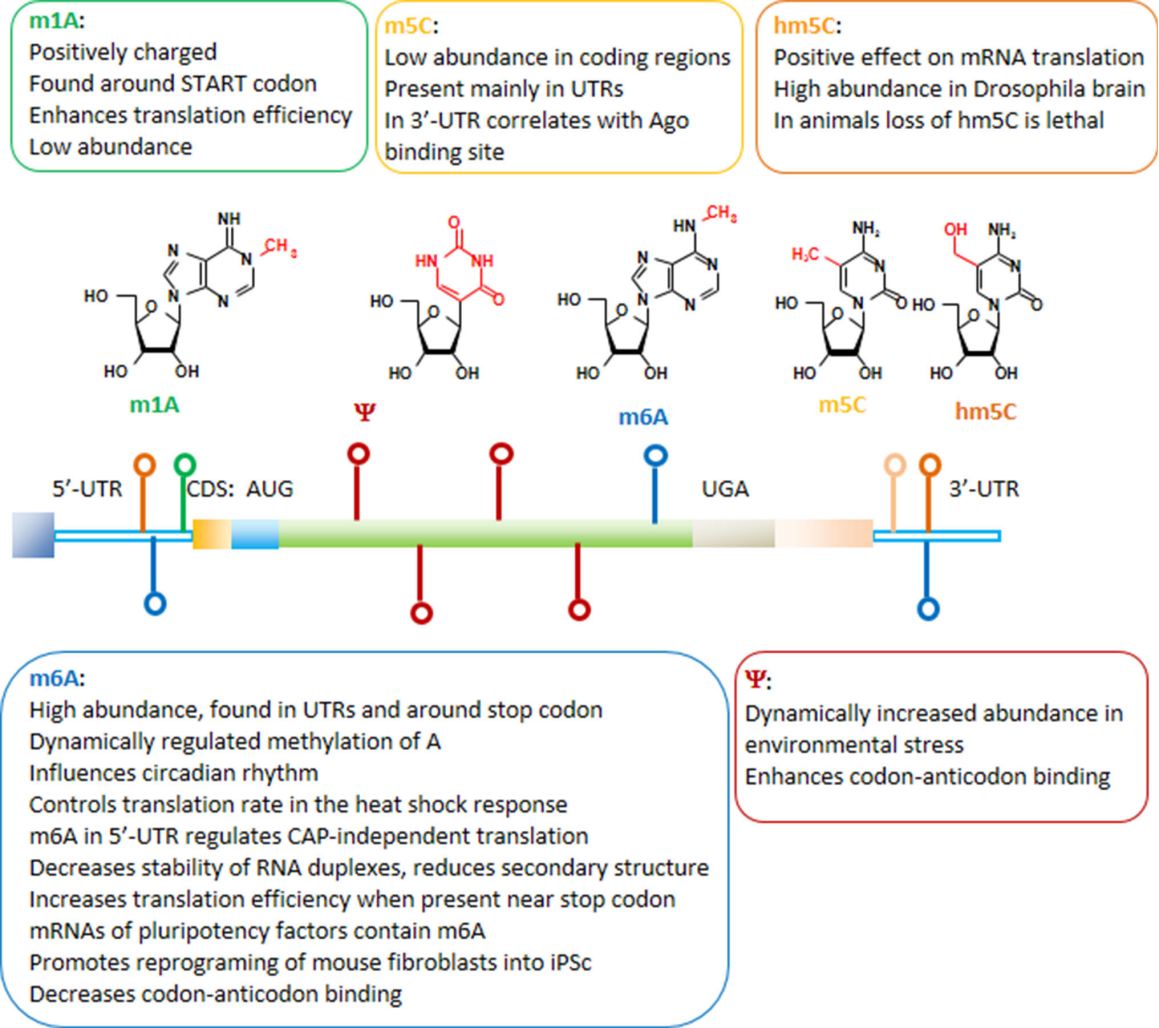
mRNA modifications and their functions. 5′- and 3′-untranslated regions (UTR), as well as start (AUG) and stop (UGA) codons are indicated. The colors of the marks along the mRNA correspond to the specific modified nucleosides

Nucleoside modifications identified in mRNA include *N*6-methyladenosine (m6A), *N*1-methyladenosine (m1A), 5-methylcytidine (m5C), 5-hydroxymethylcytosine (hm5C), and pseudouridine (Ψ) (Fig. 6) [5, 181–188]. Naturally, m6A is the most abundant modification found in mRNAs and also in non-coding RNAs [189, 190]. The m6A modifications in mRNA are preferentially found in coding sequences around the stop codon and in 3′-untranslated regions [191]. Through its influence on mRNA processing, m6A was shown to influence circadian rhythms [192]. In mammalian cells, METTL3 and METTL4 were identified as the methyltransferases modifying adenosine, and the methyl group can be removed from m6A, e.g. by FTO or ALKBH5 demethylases [193–195]. The identification of m6A demethylases suggests a dynamic regulation of adenosine methylation [196]. As a reader of the information encoded by the m6A modification, the YTH domain-containing family protein 2 (YTHDF2) was identified [197, 198]. Very recently, dynamic m6A methylation was described to control translation rates in the heat shock response [199]. During the heat shock, YTHDF2 is relocated from the cytoplasm to the nucleus and the methylation of a few adenosines within the 5′-UTR of newly transcribed mRNAs is increased. Translation initiation of these mRNAs becomes independent from the 5′-CAP structure [199]. The 5′-CAP-independent translation depends on ribosome binding inside the 5′-UTR that can be mediated by eIF3 binding to a single m6A [200]. Dynamic alterations in the amount of m6A in 5′UTR during stress conditions enable CAP-independent translation and answer a long-standing question about the mechanism of selective protein synthesis when CAP-dependent translation is suppressed. In other publications, YTHDF2 was described to promote relocation and degradation of m6A containing mRNAs [185, 197]. In general, the m6A modification destabilizes RNA duplexes and reduces secondary structure [201]. Alterations in the secondary structure by the presence of m6A can increase the accessibility of RNA for the binding of other proteins such as heterogeneous nuclear ribonucleoprotein C (HNRNPC), a nuclear protein involved in pre-mRNA processing [198], or YTHDF1, which increases translation efficiency of mRNAs containing m6A near stop codons [202]. Among the mRNAs containing conserved m6A modifications are transcripts encoding pluripotency factors [203]. Lack of m6A after depletion of Mettl3 was shown to prevent the differentiation of pluripotent stem cells into specific lineages and to preserve the pluripotent stem cell status [203, 204]. The results were confirmed by a recent study showing that the reprogramming of mouse embryonic fibroblasts into pluripotent stem cells was promoted by m6A [205].

As m6A was found in coding regions of mRNA it may exert a direct influence on codon–anticodon interactions. As demonstrated very recently, the presence of m6A within a codon decelerates cognate-tRNA decoding [206]. The influence of mRNA base modifications on decoding was further analyzed in an experimental system in which methylation of guanosine was introduced at different codon positions in mRNAs. O6-methylguanosine (m6G) in the first and third codon position severely impaired correct tRNA selection, while the same modification at the second position did not show erroneous coding but a more than 1000-fold decrease in translation rate [207]. Quantitative mass spectrometry of mammalian mRNA preparations identified *N* ^1^-methyladenosine (m1A) with an overall frequency of 0.015–0.16 % of all adenosines [208]. Under physiological conditions, this modification introduces a positive charge which is expected to strongly affect the decoding process. m1A was preferentially found around start codons and its presence increased translation efficiency [208].

In addition, studies carried out on human and yeast transcriptomes identified hundreds of pseudouridine (Ψ) sites in mRNA [183, 184, 209]. The studies suggest that mRNA pseudouridylation is an ancient, evolutionarily conserved mechanism. In non-coding RNAs, Ψ is one of the most widespread post-transcriptional modifications, highly conserved among species. The site-specific pseudouridylation of eukaryotic mRNAs is a genetically programmed RNA modification that naturally occurs in multiple transcripts via distinct mechanisms providing important regulatory functions [210]. Further research is required to identify the pseudouridine “readers”, proteins that specifically bind to Ψ sites on the mRNA. The development of a novel sequencing methodology allowing to identify Ψ in non-coding RNA at a single nucleotide level revealed that the number of Ψ sites may dynamically increase in environmental stress conditions (heat shock stimuli, nutrient deprivation or serum starvation) [183, 184, 209]. In an experimental approach, pseudouridylation targeted to uridines at the first position in stop codons was shown to suppress translation termination both in vitro and in vivo [211]. The translation termination codons were converted into coding triplets demonstrating once more the fundamental influence of nucleoside modifications on the codon recognition.

The 5-methylcytidine (m5C) mRNA base modification shows low abundance in coding regions and is found mainly in untranslated regions [212]. A function in miRNA targeting was suggested for m5C as its occurrence in the 3′-UTR correlates with Argonaute binding sites. m5C can be oxidized by Tet enzymes to the hydroxymethylcytosine nucleoside which has a positive effect on mRNA translation [188]. Investigations in Drosophila revealed that the brain was especially rich in hmC, and the flies did not survive a complete loss of RNA hydroxymethylation [188].

## Nucleoside modifications in other non-coding RNA (ncRNA)

Base modifications of non-coding RNA was a rather unexplored research field until recently. Analyses using an anti-m6A-antibody followed by RNA-sequencing (RNA-seq) revealed that also a significant fraction of miRNAs contains m6A [213]. The m6A modifications mark pri-miRNAs, the precursors of pre-miRNAs, for proper processing by the DROSHA ribonuclease and DGCR8 [214]. In addition, the abundance of miRNAs was controlled by m6A as knock-down of the m6A demethylase FTO affected the levels of some miRNAs [213]. Conversely, the formation of m6A in mRNA was shown to be regulated by miRNAs through a sequence pairing mechanism. Over-expression of certain miRNAs significantly increased the m6A abundance at the miRNA binding sites by a mechanism including the interaction of the miRNA processing enzyme Dicer with METTL3 [215]. In nuclear transcripts, m6A is part of a consensus motif which is recognized by the HNRNPA2B1 RNA binding protein to regulate alternative splicing [213]. The regulation of the miRNA pathway and the alternative splicing events by modified nucleotides adds new layers of complexity to the posttranscriptional regulation of gene expression.

## Delayed translation adversely affects cell functions

During the last 2 years, the consequences of restrained peptide bond formation caused by low abundance of specific tRNAs or aberrations in tRNA modifications were clarified. In 2014, O’Brian and colleagues found a correlation between translation speed and the correct folding of the nascent peptide chains [216, 217]. The translation speed, the rate with which amino acids are added to the growing peptide chain, depends on the codon usage in the mRNA, codons binding abundant tRNAs are processed faster [218, 219]. Strikingly, a more rapid translation facilitated the right peptide folding so that the frequency of a particular codon and the concentration of its cognate tRNA exerted an influence on the quality of the protein production.

In 2015, Nedialkova and Leidel confirmed and expanded these findings by showing that ribosome pausing at codons with low abundancy of their cognate tRNAs increased protein misfolding and aggregation which eventually resulted in impaired protein homeostasis and cellular dysfunction [220]. The ribosome pausing had been demonstrated earlier by determination of the in vivo ribosome distribution on the mRNA at single codon resolution [221]. Deng et al. demonstrated that enhanced ribosome pausing in yeast facilitates down-regulation of the expressed proteins [222]. It was also suggested that the existence of a ‘codon ramp’, a stretch of 30–50 codons enriched with suboptimal codons, slows down translation to avoid downstream crowding of ribosomes [223]. However, the influence of translation speed seems to affect more functional features of proteins including the microdomain structures of intrinsically disordered regions [224, 225]. An unusual example for non-optimal codon usage required for a functioning circadian rhythm has been described in *Neurospora*. Codon optimization for the clock protein FRQ not only increased the protein level and disturbed the circadian rhythms, but also resulted in conformational changes in the FRQ protein [26]. The speed of ribosome movement can even exert a kind of feedback control on the translation initiation efficiency [226]. Consequently, tRNA modifications and their dynamic changes influence the translation elongation rate to control not only protein levels but also the quality of protein folding.

## Conclusion

To prevent the simultaneous execution incompatible cellular programs the schedule of gene expression has to be harmonized within a reasonable response time. This control includes the adjustment of the concentration and stability of mRNAs as executed by transcription regulation, splicing, nuclear export, RNA interference, etc. to subsequently favor the synthesis of required proteins while decreasing the production of actually not needed ones. Furthermore, mechanisms are required during life-threatening events to immediately shut down overall protein production and to concentrate the cells’ capacity on the synthesis of rescue proteins able to ease the stressful conditions. The tRNA molecules have evolved as stress sensors and mediators of immediate early stress response pathways due to their central role in protein translation. tRNAs and tRNA-derived fragments contribute to the control of apoptosis, stress signaling, and RNA interference. The presence or absence of tRNA nucleoside modifications modulate these processes by (1) controlling enzymatic tRNA cleavage determining the concentration of tRNA halves with stress signaling function, and (2) influencing translation fidelity to promote or ignore specific codons resulting in biased translation of mRNAs. The redundancy of the genetic code allows for such preference shifts in codon usage and that is why position 34 in tRNAs, the wobble nucleoside, is a common target for translation-altering modifications. A bewildering number of tRNA base variations have been described and with high probability more are waiting to be discovered.

## References

[1] Palstra RJ, Grosveld F (2012). Transcription factor binding at enhancers: shaping a genomic regulatory landscape in flux. Front Genet.

[2] Mili S, Shu HJ, Zhao Y, Piñol-Roma S (2001). Distinct RNP complexes of shuttling hnRNP proteins with pre-mRNA and mRNA: candidate intermediates in formation and export of mRNA. Mol Cell Biol.

[3] Nagai K, Muto Y, Pomeranz Krummel DA, Kambach C, Ignjatovic T, Walke S, Kuglstatter A (2001). Structure and assembly of the spliceosomal snRNPs. Novartis Medal Lecture. Biochem Soc Trans.

[4] Jia J, Yao P, Arif A, Fox PL (2013). Regulation and dysregulation of 3′UTR-mediated translational control. Curr Opin Genet Dev.

[5] Meyer KD, Jaffrey SR (2014). The dynamic epitranscriptome: *N*6-methyladenosine and gene expression control. Nat Rev Mol Cell Biol.

[6] Yuan S, Tang H, Xing J, Fan X, Cai X, Li Q, Han P, Luo Y, Zhang Z, Jiang B, Dou Y, Gorospe M, Wang W (2014). Methylation by NSun2 represses the levels and function of microRNA 125b. Mol Cell Biol.

[7] Pichon X, Wilson LA, Stoneley M, Bastide A, King HA, Somers J, Willis AE (2012). RNA binding protein/RNA element interactions and the control of translation. Curr Protein Pept Sci.

[8] Grewal SS (2015). Why should cancer biologists care about tRNAs? tRNA synthesis, mRNA translation and the control of growth. Biochim Biophys Acta.

[9] Björk GR, Hagervall TG (2014). Transfer RNA modification: presence, synthesis, and function. EcoSal Plus.

[10] Banerjee R, Chen S, Dare K, Gilreath M, Praetorius-Ibba M, Raina M, Reynolds NM, Rogers T, Roy H, Yadavalli SS, Ibba M (2010). tRNAs: cellular barcodes for amino acids. FEBS Lett.

[11] Nawrot B, Sochacka E, Düchler M (2011). tRNA structural and functional changes induced by oxidative stress. Cell Mol Life Sci.

[12] Lee YS, Shibata Y, Malhotra A, Dutta A (2009). A novel class of small RNAs: tRNA-derived RNA fragments (tRFs). Genes Dev.

[13] Shigematsu M, Kirino Y (2015). tRNA-derived short non-coding RNA as interacting partners of argonaute proteins. Gene Regul Syst Bio.

[14] Maute RL, Schneider C, Sumazin P, Holmes A, Califano A, Basso K, Dalla-Favera R (2013). tRNA-derived microRNA modulates proliferation and the DNA damage response and is down-regulated in B cell lymphoma. Proc Natl Acad Sci USA.

[15] Martens-Uzunova ES, Olvedy M, Jenster G (2013). Beyond microRNA–novel RNAs derived from small non-coding RNA and their implication in cancer. Cancer Lett.

[16] Yeung ML, Bennasser Y, Watashi K, Le SY, Houzet L, Jeang KT (2009). Pyrosequencing of small non-coding RNAs in HIV-1 infected cells: evidence for the processing of a viral-cellular double-stranded RNA hybrid. Nucleic Acids Res.

[17] Machnicka MA, Milanowska K, Osman Oglou O, Purta E, Kurkowska M, Olchowik A, Januszewski W, Kalinowski S, Dunin-Horkawicz S, Rother KM, Helm M, Bujnicki JM, Grosjean H (2013). MODOMICS: a database of RNA modification pathways—2013 update. Nucleic Acids Res.

[18] Cantara WA, Crain PF, Rozenski J, McCloskey JA, Harris KA, Zhang X, Vendeix FA, Fabris D, Agris PF (2011). The RNA modification database, RNAMDB: 2011 update. Nucleic Acids Res.

[19] Madore E, Florentz C, Giegé R, Sekine S, Yokoyama S, Lapointe J (1999). Effect of modified nucleotides on Escherichia coli tRNAGlu structure and on its aminoacylation by glutamyl-tRNA synthetase. Predominant and distinct roles of the mnm5 and s2 modifications of U34. Eur J Biochem.

[20] Klassen R, Paluszynski JP, Wemhoff S, Pfeiffer A, Fricke J, Meinhardt F (2008). The primary target of the killer toxin from Pichia acaciae is tRNA(Gln). Mol Microbiol.

[21] Saikia M, Krokowski D, Guan BJ, Ivanov P, Parisien M, Hu GF, Anderson P, Pan T, Hatzoglou M (2012). Genome-wide identification and quantitative analysis of cleaved tRNA fragments induced by cellular stress. J Biol Chem.

[22] Schaefer M, Pollex T, Hanna K, Tuorto F, Meusburger M, Helm M, Lyko F (2010). RNA methylation by Dnmt2 protects transfer RNAs against stress-induced cleavage. Genes Dev.

[23] Blanco S, Dietmann S, Flores JV, Hussain S, Kutter C, Humphreys P, Lukk M, Lombard P, Treps L, Popis M, Kellner S, Hölter SM, Garrett L, Wurst W, Becker L, Klopstock T, Fuchs H, Gailus-Durner V, Hrabĕ de Angelis M, Káradóttir RT, Helm M, Ule J, Gleeson JG, Odom DT, Frye M (2014). Aberrant methylation of tRNAs links cellular stress to neuro-developmental disorders. EMBO J.

[24] Manickam N, Joshi K, Bhatt  MJ, Farabaugh PJ (2016). Effects of tRNA modification on translational accuracy depend on intrinsic codon–anticodon strength. Nucleic Acids Res.

[25] Hori H (2014). Methylated nucleosides in tRNA and tRNA methyltransferases. Front Genet.

[26] Zhou M, Guo J, Cha J, Chae M, Chen S, Barral JM, Sachs MS, Liu Y (2013). Non-optimal codon usage affects expression, structure and function of clock protein FRQ. Nature.

[27] Mami I, Pallet N (2015) Transfer RNA fragmentation and protein translation dynamics in the course of kidney injury. RNA Biol doi:10.1080/15476286.2015.1107704 10.1080/15476286.2015.1107704 PMC6284587 26513712

[28] Fu H, Feng J, Liu Q, Sun F, Tie Y, Zhu J, Xing R, Sun Z, Zheng X (2009). Stress induces tRNA cleavage by angiogenin in mammalian cells. FEBS Lett.

[29] Yamasaki S, Ivanov P, Hu GF, Anderson P (2009). Angiogenin cleaves tRNA and promotes stress-induced translational repression. J Cell Biol.

[30] Czech A, Wende S, Mörl M, Pan T, Ignatova Z (2013). Reversible and rapid transfer-RNA deactivation as a mechanism of translational repression in stress. PLoS Genet.

[31] Emara MM, Ivanov P, Hickman T, Dawra N, Tisdale S, Kedersha N, Hu GF, Anderson P (2010). Angiogenin-induced tRNA-derived stress-induced RNAs promote stress-induced stress granule assembly. J Biol Chem.

[32] Pizzo E, Sarcinelli C, Sheng J, Fusco S, Formiggini F, Netti P, Yu W, D’Alessio G, Hu GF (2013). Ribonuclease/angiogenin inhibitor 1 regulates stress-induced subcellular localization of angiogenin to control growth and survival. J Cell Sci.

[33] Buchan JR (2013). Eukaryotic stress granules are cleared by autophagy and Cdc48/VCP function. Cell.

[34] Tomita K, Ogawa T, Uozumi T, Watanabe K, Masaki H (2000). A cytotoxic ribonuclease which specifically cleaves four isoaccepting arginine tRNAs at their anticodon loops. Proc Natl Acad Sci USA.

[35] Meineke B, Schwer B, Schaffrath R, Shuman S (2011). Determinants of eukaryal cell killing by the bacterial ribotoxin PrrC. Nucleic Acids Res.

[36] Shigematsu M, Ogawa T, Kitamoto HK, Hidaka M, Masaki H (2012). Specific phase arrest of cell cycle restores cell viability against tRNA cleavage by killer toxin. Biochem Biophys Res Commun.

[37] Saxena SK, Sirdeshmukh R, Ardelt W, Mikulski SM, Shogen K, Youle RJ (2002). Entry into cells and selective degradation of tRNAs by a cytotoxic member of the RNase A family. J Biol Chem.

[38] Meineke B, Kast A, Schwer B, Meinhardt F, Shuman S, Klassen R (2012). A fungal anticodon nuclease ribotoxin exploits a secondary cleavage site to evade tRNA repair. RNA.

[39] Tuorto F, Liebers R, Musch T, Schaefer M, Hofmann S, Kellner S, Frye M, Helm M, Stoecklin G, Lyko F (2012). RNA cytosine methylation by Dnmt2 and NSun2 promotes tRNA stability and protein synthesis. Nat Struct Mol Biol.

[40] Mishima E, Inoue C, Saigusa D, Inoue R, Ito K, Suzuki Y (2014). Conformational change in transfer RNA is an early indicator of acute cellular damage. J Am Soc Nephrol.

[41] Ivanov P, Emara MM, Villen J, Gygi SP, Anderson P (2011). Angiogenin-induced tRNA fragments inhibit translation initiation. Mol Cell.

[42] Dhahbi JM, Spindler SR, Atamna H, Yamakawa A, Boffelli D, Mote P, Martin DI (2013). 5′ tRNA halves are present as abundant complexes in serum, concentrated in blood cells, and modulated by aging and calorie restriction. BMC Genom.

[43] Goodarzi H, Liu X, Nguyen HC, Zhang S, Fish L, Tavazoie SF (2015). Endogenous tRNA-derived fragments suppress breast cancer progression via YBX1 displacement. Cell.

[44] Casas E, Cai G, Neill JD (2015). Characterization of circulating transfer RNA-derived RNA fragments in cattle. Front Genet.

[45] Telonis AG, Loher P, Honda S, Jing Y, Palazzo J, Kirino Y, Rigoutsos I (2015). Dissecting tRNA-derived fragment complexities using personalized transcriptomes reveals novel fragment classes and unexpected dependencies. Oncotarget.

[46] Mei Y, Yong J, Liu H, Shi Y, Meinkoth J, Dreyfuss G, Yang X (2010). tRNA binds to cytochrome c and inhibits caspase activation. Mol Cell.

[47] Saikia M, Jobava R, Parisien M, Putnam A, Krokowski D, Gao XH, Guan BJ, Yuan Y, Jankowsky E, Feng Z, Hu GF, Pusztai-Carey M, Gorla M, Sepuri NB, Pan T, Hatzoglou M (2014). Angiogenin-cleaved tRNA halves interact with cytochrome c, protecting cells from apoptosis during osmotic stress. Mol Cell Biol.

[48] Li Q, Hu B, Hu GW, Chen CY, Niu X, Liu J, Zhou SM, Zhang CQ, Wang Y, Deng ZF (2016). tRNA-derived small non-coding RNAs in response to ischemia inhibit angiogenesis. Sci Rep.

[49] Honda S, Loher P, Shigematsu M, Palazzo JP, Suzuki R, Imoto I, Rigoutsos I, Kirino Y (2015). Sex hormone-dependent tRNA halves enhance cell proliferation in breast and prostate cancers. Proc Natl Acad Sci USA.

[50] Lopes RR, Kessler AC, Polycarpo C, Alfonzo JD (2015). Cutting, dicing, healing and sealing: the molecular surgery of tRNA. Wiley Interdiscip Rev RNA.

[51] Kanai A (2015). Disrupted tRNA genes and tRNA fragments: a perspective on tRNA gene evolution. Life (Basel).

[52] Torres AG, Batlle E, Ribas de Pouplana L (2014). Role of tRNA modifications in human diseases. Trends Mol Med.

[53] Guy MP, Shaw M, Weiner CL, Hobson L, Stark Z, Rose K, Kalscheuer VM, Gecz J, Phizicky EM (2015). Defects in tRNA anticodon loop 2′-*O*-methylation are implicated in nonsyndromic X-linked intellectual disability due to mutations in FTSJ1. Hum Mutat.

[54] Guy MP, Phizicky EM (2015). Conservation of an intricate circuit for crucial modifications of the tRNAPhe anticodon loop in eukaryotes. RNA.

[55] Karlsborn T, Tükenmez H, Chen C, Byström AS (2014). Familial dysautonomia (FD) patients have reduced levels of the modified wobble nucleoside mcm(5)s(2)U in tRNA. Biochem Biophys Res Commun.

[56] Thiaville PC, de Crecy-Lagard V (2015). The emerging role of complex modifications of tRNALysUUU in signaling pathways. Microb Cell.

[57] Wilusz JE (2015). Controlling translation via modulation of tRNA levels. Wiley Interdiscip Rev RNA.

[58] Agris PF, Vendeix FA, Graham WD (2007). tRNA’s wobble decoding of the genome: 40 years of modification. J Mol Biol.

[59] Krisko A, Copic T, Gabaldón T, Lehner B, Supek F (2014). Inferring gene function from evolutionary change in signatures of translation efficiency. Genome Biol.

[60] Begley U, Dyavaiah M, Patil A, Rooney JP, Direnzo D (2007). Trm9-catalyzed tRNA modifications link translation to the DNA damage response. Mol Cell.

[61] Brandmayr C, Wagner M, Brückl T, Globisch D, Pearson D, Kneuttinger AC, Reiter V, Hienzsch A, Koch S, Thoma I, Thumbs P, Michalakis S, Müller M, Biel M, Carell T (2012). Isotope-based analysis of modified tRNA nucleosides correlates modification density with translational efficiency. Angew Chem Int Ed Engl.

[62] Rezgui VA, Tyagi K, Ranjan N, Konevega AL, Mittelstaet J, Rodnina MV, Peter M, Pedrioli PG (2013). tRNA tKUUU, tQUUG, and tEUUC wobble position modifications fine-tune protein translation by promoting ribosome A-site binding. Proc Natl Acad Sci USA.

[63] Tükenmez H, Xu H, Esberg A, Byström AS (2015). The role of wobble uridine modifications in +1 translational frameshifting in eukaryotes. Nucleic Acids Res.

[64] Lamichhane TN, Blewett NH, Crawford AK, Cherkasova VA, Iben JR, Begley TJ, Farabaugh PJ, Maraia RJ (2013). Lack of tRNA modification isopentenyl-A37 alters mRNA decoding and causes metabolic deficiencies in fission yeast. Mol Cell Biol.

[65] Miyauchi K, Kimura S, Suzuki T (2013). A cyclic form of *N*6-threonylcarbamoyladenosine as a widely distributed tRNA hypermodification. Nat Chem Biol.

[66] Matuszewski M, Sochacka E (2014). Stability studies on the newly discovered cyclic form of tRNA *N*(6)-threonylcarbamoyladenosine (ct(6)A). Bioorg Med Chem Lett.

[67] Crick FHC (1966). Codon–anticodon pairing: the wobble hypothesis. J Mol Biol.

[68] Sylvers LA, Rogers KC, Shimizu M, Ohtsuka E, Söll D (1993). A 2-thiouridine derivative in tRNAGlu is a positive determinant for aminoacylation by *Escherichia coli* glutamyl-tRNA synthetase. Biochemistry.

[69] Karikó K, Buckstein M, Ni H, Weissman D (2005). Suppression of RNA recognition by Toll-like receptors: the impact of nucleoside modification and the evolutionary origin of RNA. Immunity.

[70] Nallagatla SR, Bevilacqua PC (2008). Nucleoside modifications modulate activation of the protein kinase PKR in an RNA structure-specific manner. RNA.

[71] Nallagatla SR, Jones CN, Ghosh SK, Sharma SD, Cameron CE, Spremulli LL, Bevilacqua PC (2013). Native tertiary structure and nucleoside modifications suppress tRNA’s intrinsic ability to activate the innate immune sensor PKR. PLoS One.

[72] Isel C, Marquet R, Keith G, Ehresmann C, Ehresmann B (1993). Modified nucleotides of tRNA(3Lys) modulate primer/template loop-loop interaction in the initiation complex of HIV-1 reverse transcription. J Biol Chem.

[73] Watanabe K, Oshima T, Saneyoshi M, Nishimura S (1974). Replacement of ribothymidine by 5-methyl-2-thiouridine in sequence GT psi C in tRNA of an extreme thermophile. FEBS Lett.

[74] Sprinzl M, Horn C, Brown M, Ioudovitch A, Steinberg S (1998). Compilation of tRNA sequences and sequences of tRNA genes. Nucleic Acids Res.

[75] Kowalak JA, Dalluge JJ, McCloskey JA, Stetter KO (1994). The role of posttranscriptional modification in stabilization of transfer RNA from hyperthermophiles. Biochemistry.

[76] Laxman S, Sutter BM, Wu X, Kumar S, Guo X, Trudgian DC, Mirzaei H, Tu BP (2013). Sulfur amino acids regulate translational capacity and metabolic homeostasis through modulation of tRNA thiolation. Cell.

[77] Han L, Kon Y, Phizicky EM (2015). Functional importance of Ψ38 and Ψ39 in distinct tRNAs, amplified for tRNAGln(UUG) by unexpected temperature sensitivity of the s2U modification in yeast. RNA.

[78] Yarian C, Townsend H, Czestkowski W, Sochacka E, Malkiewicz AJ, Guenther R, Miskiewicz A, Agris PF (2002). Accurate translation of the genetic code depends on tRNA modified nucleosides. J Biol Chem.

[79] Sochacka E, Kraszewska K, Sochacki M, Sobczak M, Janicka M, Nawrot B (2011). The 2-thiouridine unit in the RNA strand is desulfured predominantly to 4-pyrimidinone nucleoside under in vitro oxidative stress conditions. Chem Commun (Camb).

[80] Sochacka E, Bartos P, Kraszewska K, Nawrot B (2013). Desulfuration of 2-thiouridine with hydrogen peroxide in the physiological pH range 6.6-7.6 is pH-dependent and results in two distinct products. Bioorg Med Chem Lett.

[81] Bartos P, Ebenryter-Olbinska K, Sochacka E, Nawrot B (2015). The influence of the C5 substituent on the 2-thiouridine desulfuration pathway and the conformational analysis of the resulting 4-pyrimidinone products. Bioorg Med Chem.

[82] Sochacka E, Szczepanowski RH, Cypryk M, Sobczak M, Janicka M, Kraszewska K, Bartos P, Chwialkowska A, Nawrot B (2015). 2-Thiouracil deprived of thiocarbonyl function preferentially base pairs with guanine rather than adenine in RNA and DNA duplexes. Nucleic Acids Res.

[83] Srinivasan T, Kumaran K, Selvakumar R, Velmurugan D, Sudarsanam D (2013). Exploring GpG bases next to anticodon in tRNA subsets. Bioinformation.

[84] Tuorto F, Herbst F, Alerasool N, Bender S, Popp O, Federico G, Reitter S, Liebers R, Stoecklin G, Gröne HJ, Dittmar G, Glimm H, Lyko F (2015). The tRNA methyltransferase Dnmt2 is required for accurate polypeptide synthesis during haematopoiesis. EMBO J.

[85] Björk GR, Huang B, Persson OP, Byström AS (2007). A conserved modified wobble nucleoside (mcm5s2U) in lysyl-tRNA is required for viability in yeast. RNA.

[86] D’Silva S, Haider SJ, Phizicky EM (2011). A domain of the actin binding protein Abp140 is the yeast methyltransferase responsible for 3-methylcytidine modification in the tRNA anticodon loop. RNA.

[87] Haag S, Warda AS, Kretschmer J, Günnigmann MA, Höbartner C, Bohnsack MT (2015). NSUN6 is a human RNA methyltransferase that catalyzes formation of m5C72 in specific tRNAs. RNA.

[88] Pietromonaco S, Denslow N, O‘Brien TW (1991). Proteins of mammalian mitochondrial ribosomes. Biochimie.

[89] Christian BE, Spremulli LL (2012). Mechanism of protein biosynthesis in mammalian mitochondria. Biochim Biophys Acta.

[90] Boczonadi V, Horvath R (2014). Mitochondria: impaired mitochondrial translation in human disease. Int J Biochem Cell Biol.

[91] Salinas-Giegé T, Giegé P (2015). tRNA biology in mitochondria. Int J Mol Sci.

[92] Powell ChA, Nicholls TJ, Minczuk M (2015). Nuclear-encoded factors involved in post-transcriptional processing and modification of mitochondrial tRNAs in human disease Front Gen.

[93] Suzuki T, Nagao A, Suzuki T (2011). Human mitochondrial tRNAs: biogenesis, function, structural aspects, and diseases. Annu Rev Genet.

[94] Watanabe K, Yokobori S (2011). tRNA modification and genetic code variations in animal mitochondria. J Nucleic Acids.

[95] Suzuki T, Suzuki T (2014). A complete landscape of post-transcriptional modifications in mammalian mitochondrial tRNAs. Nucleic Acids Res.

[96] Machnicka MA, Olchowik A, Grosjean H, Bujnicki JM (2014). Distribution and frequencies of post-transcriptional modifications in tRNAs. RNA Biol.

[97] Helm M, Brulé H, Degoul F, Cepanec C, Leroux J-P, Giegé R, Florentz C (1998). The presence of modified nucleotides is required for cloverleaf folding of a human mitochondrial tRNA. Nucleic Acids Res.

[98] Jones CI, Spencer AC, Hsu JL, Spremulli LL, Martinis SA, DeRider M, Agris PF (2006). A Counterintuitive Mg2+-dependent and modification-assisted functional folding of mitochondrial tRNAs. J Mol Biol.

[99] Bhaskaran H, Taniguchi T, Suzuki T, Suzuki T, Perona JJ (2014). Structural dynamics of a mitochondrial tRNA possessing weak thermodynamic stability. Biochemistry.

[100] Helm M, Giege R, Florentz C (1999). A Watson–Crick base-pair-disrupting methyl group (m1A9) is sufficient for cloverleaf folding of human mitochondrial tRNALys. Biochemistry.

[101] Voigts-Hoffmann F, Hengesbach M, Kobitski AY, van Aerschot A, Herdewijn P, Nienhaus GU, Helm M (2007). A methyl group controls conformational equilibrium in human mitochondrial tRNALys. J Am Chem Soc.

[102] Moraes CT, Ciacci F, Bonilla E, Ionasescu V, Schon EA, Dimauro S (1993). A mitochondrial transfer-RNA anticodon swap associated with a muscle disease. Nat Genet.

[103] Enriquez JA, Chomyn A, Attardi G (1995). mtDNA mutation in MERRF-syndrome causes defective aminoacylation of tRNALys and premature translation termination. Nat Genet.

[104] Armengod NE, Meseguer S, Villarroya M, Prado S, Moukadiri I, Ruiz-Partida R, Garzon MJ, Navarro-Gonzalez C, Matinez-Zamora A (2014). Modification of the wobble uridine in bacterial and mitochondrial tRNAs reading NNA/NNG triplets of 2-codon boxes. RNA Biol.

[105] Yasukawa T, Suzuki T, Ishii N, Ueda T, Ohta S, Watanabe K (2000). Defect in modification at the anticodon wobble nucleotide of mitochondrial tRNALys with the MERRF encephalomyopathy pathogenic mutation. FEBS Lett.

[106] Suzuki T, Suzuki T, Wada T, Saigo K, Watanabe K (2001). Novel taurine-containing derivatives and mitochondrial human diseases. Nucleic Acids Res.

[107] Moriya J, Yokogawa T, Wakita K, Ueda T, Nishikawa K, Crain PF, Hashizume T, Pomerantz SC, McCloskey JA, Kawai G (1994). A novel modified nucleoside found at the first position of the anticodon of methionine tRNA from bovine liver mitochondria. Biochemistry.

[108] Suzuki T, Nagao A, Suzuki T (2011). Human mitochondrial diseases caused by lack of taurine modification in mitochondrial tRNAs. Wiley Interdiscip Rev RNA.

[109] Levinger L, Morl M, Florentz C (2004). Mitochondrial tRNA 3′ end metabolism and human disease. Nucleic Acids Res.

[110] Belostotsky R, Frishberg Y, Entelis N (2012). Human mitochondrial tRNA quality control in health and disease: a channelling mechanism?. RNA Biol.

[111] Shoffner JM, Lott MT, Lezza AM, Seibel P, Ballinger SW, Wallace DC (1990). Myoclonic epilepsy and ragged-red fiber disease (MERRF) is associated with a mitochondrial DNA tRNA(Lys) mutation. Cell.

[112] Lightowlers RN, Taylor RW, Turnbull DM (2015). Mutations causing mitochondrial disease: what is new challenges remain?. Science.

[113] Goto Y, Nonaka I, Horai S (1990). A mutation in the tRNA(Leu)(UUR) gene associated with the MELAS subgroup of mitochondrial encephalomyopathies. Nature.

[114] Goto Y, Nonaka I, Horai S (1991). A new mtDNA mutation associated with mitochondrial myopathy, encephalopathy, lactic acidosis and stroke-like episodes (MELAS). Biochim Biophys Acta.

[115] Yasukawa T, Suzuki T, Suzuki T, Ueda T, Ohta S, Watanabe K (2000). Modification defect at anticodon wobble nucleotide of mitochondrial tRNAsLeu(UUR) with pathogenic mutations of mitochondrial myopathy, encephalopathy, lactic acidosis, and stroke-like episodes. J Biol Chem.

[116] Kirino Y, Goto Y, Campos Y, Arenas J, Suzuki T (2005). Specific correlation between the wobble modification deficiency in mutant tRNAs and the clinical features of a human mitochondrial disease. Proc Natl Acad Sci USA.

[117] Yasukawa T, Kirino Y, Ishii N, Holt IJ, Jacobs HT, Makifuchi T, Fukuhara N, Ohta S, Suzuki T, Watanabe K (2005). Wobble modification deficiency in mutant tRNAs in patients with mitochondrial diseases. FEBS Lett.

[118] Kurata S, Weixlbaumer A, Ohtsuki T, Shimazaki T, Wada T, Kirino Y, Takai K, Watanabe K, Ramakrishnan V, Suzuki T (2008). Modified uridines with C5-methylene substituents at the first position of the tRNA anticodon stabilize U•G wobble pairing during decoding. J Biol Chem.

[119] Rodriguez-Hernandez A, Spears JL, Gaston KW, Limbach PA, Gamper H, Hou YM, Kaiser R, Agris PF, Perona JJ (2013). Structural and mechanistic basis for enhanced translational efficiency by 2-thiouridine at the tRNA anticodon wobble position. J Mol Biol.

[120] Ashraf SS, Sochacka E, Cain R, Guenther R, Malkiewicz A, Agris PF (1999). Single atom modification (O → S) of tRNA confers ribosome binding. RNA.

[121] Yasukawa T, Suzuki T, Ishii N, Ohta S, Watanabe K (2001). Wobble modification defect in tRNA disturbs codon–anticodon interaction in a mitochondrial disease. EMBO J.

[122] Wang X, Yan Q, Guan MX (2010). Combination of the loss of cmnm5U34 with the lack of s2U34 modifications of tRNALys, tRNAGlu, and tRNAGln altered mitochondrial biogenesis and respiration. J Mol Biol.

[123] Schara U, von Kleist-Retzow JC, Lainka E, Gerner P, Pyle A, Smith PM, Lochmuller H, Czermin B, Abicht A, Holinski-Feder E, Horvath R (2011). Acute liver failure with subsequent cirrhosis as the primary manifestation of TRMU mutations. J Inherit Metab Dis.

[124] Ghezzi D, Baruffini E, Haack TB, Invernizzi F, Melchionda L, Dallabona C, Strom TM, Parini R, Burlina AB, Meitinger T, Prokisch H, Ferrero I, Zeviani M (2012). Mutations of the mitochondrial-tRNA modifier MTO1 cause hypertrophic cardiomyopathy and lactic acidosis. Am J Hum Genet.

[125] Tischner Ch, Hofer A, Wulff V, Stepek J, Dumitru I, Becker L, Haak T, Kremer L, Datta AN, Sperl W, Floss T, Wurst W, Chrzanowska-Lightowlers Z, Hrabe de Angelis M, Klopstock T, Prokisch H, Wenz T (2015). MTO1 mediates tissue-specificity of OXPHOS defects via tRNA modification and translation optimization, which can be bypassed by dietary intervention. Hum Mol Genet.

[126] Sasarman F, Antonicka H, Horvath R, Shoubridge EA (2011). The 2-thiouridylase function of the human MTU1 (TRMU) enzyme is dispensable for mitochondrial translation. Hum Mol Genet.

[127] Boczonadi V, Smith PM, Pyle A, Gomez-Duran A, Schara U, Tulinus M, Chinnery PF, Horvath R (2013). Altered 2-thiouridylation impairs mitochondrial translation in reversible infantile respiratory chain deficiency. Hum Mol Genet.

[128] Takemoto C, Spremulli LL, Benkowski LA, Ueda T, Yokogawa T, Watanabe K (2009). Unconventional decoding of the AUA codon as methionine by mitochondrial tRNAMet with the anticodon f5CAU as revealed with a mitochondrial in vitro translation system. Nucleic Acids Res.

[129] BilbilleY Gustilo EM, Harris KA, Jones CN, Lusic H, Kaiser RJ, Delaney MO, Spremulli LL, Deiters A, Agris PF (2011). The human mitochondrial tRNAMet: structure/function relationship of a unique modification in the decoding of unconventional codons. J Mol Biol.

[130] Cantara WA, Murphy FV, Demirci H, Agris PF (2013). Expanded use of sense codons is regulated by modified cytidines in tRNA. Proc Natl Acad Sci USA.

[131] Yokoyama S, Miyazawa T, Iitaka Y, Yamaizumi Z, Kasai H, Nishimura S (1979). Three-dimensional structure of hypermodified nucleoside Q located in the wobbling position of tRNA. Nature.

[132] Morris RC, Brown KG, Elliott MS (1999). The effect of queuosine on tRNA structure and function. J Biomol Struct Dyn.

[133] Vinayak M, Pathak C (2010). Queuosine modification of tRNA: its divergent role in cellular machinery. Biosci Rep.

[134] Meier F, Suter B, Grosjean H, Keith G, Kubli E (1985). Queuosine modification of the wobble base in tRNAHis influences ‘in vivo’ decoding properties. EMBO J.

[135] Murphy FV, Ramakrishnan V, Malkiewicz A, Agris PF (2004). The role of modification in codon discrimination by tRNALysUUU. Nat Struct Mol Biol.

[136] Thiaville PC, El Yacoubi B, Köhrer C, Thiaville JJ, Deutsch C, Iwata-Reuyl D, Bacusmo JM, Armengaud J, Bessho Y, Wetzel C, Cao X, Limbach PA, RajBhandary UL, de Crécy-Lagard V (2015). Essentiality of threonylcarbamoyladenosine (t(6) A), a universal tRNA modification, in bacteria. Mol Microbiol.

[137] Wan LCK, Mao DYL, Neculai D, Strecker J, Chiovitti D, Kurinov I, Poda G, Thevakumaran N, Yuan F, Szilard RK, Lissina E, Nislow C, Caudy AA, Durocher D, Sicheri F (2013). Reconstitution and characterization of eukaryotic N6-threonylcarbamoylation of tRNA using a minimal enzyme system. Nucleic Acids Res.

[138] Urbonavicius J, Qian Q, Durand JM, Hagervall TG, Bjork GR (2001). Improvement of reading frame maintenance is a common function for several tRNA modifications. EMBO J.

[139] El Yacoubi B, Bailly M, de Crecy-Lagard V (2012). Biosynthesis and function of posttranscriptional modifications of transfer RNAs. Annu Rev Genet.

[140] Yarham JW, Lamichhane TN, Pyle A, Mattijssen S, Baruffini E, Bruni F, Donnini C, Vassilev A, He L, Blakely EL, Griffin H, Santibanez-Koref M, Bindoff LA, Ferrero I, Chinnery PF, McFarland R, Maraia RJ, Taylor RW (2014). Defective i6A37 modification of mitochondrial and cytosolic tRNAs results from pathogenic mutations in TRIT1 and its substrate tRNA. PLoS Genet.

[141] Reiter V, Matschkal DMS, Wagner M, Globisch D, Kneuttinger AC, Müller M, Carell T (2012). The CDK5 repressor CDK5RAP1 is a methylthiotransferase acting on nuclear and mitochondrial RNA. Nucleic Acids Res.

[142] Wei FY, Zhou B, Suzuki T, Miyata K, Ujihara Y, Horiguchi H, Takahashi N, Xie P, Michiue H, Fujimura A, Kaitsuka T, Matsui H, Koga Y, Mohri S, Suzuki T, Oike Y, Tomizawa K (2015). Cdk5rap1-mediated 2-methylthio modification of mitochondrial tRNAs governs protein translation and contributes to myopathy in mice and humans. Cell Metab.

[143] Davis DR (1995). Stabilization of RNA stacking by pseudouridine. Nucleic Acids Res.

[144] Newby MI, Greenbaum NL (2002). Investigation of overhauser effects between pseudouridine and water protons in RNA helices. Proc Natl Acad Sci USA.

[145] Charette M, Gray MW (2000). Pseudouridine in RNA: what, where, how, and why. IUBMB Life.

[146] Hamma T, Ferré-D’Amaré AR (2006). Pseudouridine synthases. Chem Biol.

[147] Bykhovskaya Y, Casas K, Mengesha E, Inbal A, Fischel-Ghodsian N (2004). Missense mutation in pseudouridine synthase 1 (PUS1) causes mitochondrial myopathy and sideroblastic anemia (MLASA). Am J Hum Genet.

[148] Fernandez-Vizarra E, Berardinelli A, Valente L, Tiranti V, Zeviani M (2007). Nonsense mutation in pseudouridylate synthase 1 (PUS1) in two brothers affected by myopathy, lactic acidosis and sideroblastic anemia (MLASA). J Med Genet.

[149] Endres L, Dedon PC, Begley TJ (2015). Codon-biased translation can be regulated by wobble-base tRNA modification systems during cellular stress responses. RNA Biol.

[150] Chan CT, Deng W, Li F, DeMott MS, Babu IR, Begley TJ, Dedon PC (2015). Highly predictive reprogramming of tRNA modifications is linked to selective expression of codon-biased genes. Chem Res Toxicol.

[151] Chan CTY, Dyavaiah M, DeMott MS, Taghizadeh K, Dedon PC (2010). A quantitative systems approach reveals dynamic control of tRNA modifications during cellular stress. PLoS Genet.

[152] Chan CT, Pang YL, Deng W, Babu IR, Dyavaiah M, Begley TJ, Dedon PC (2012). Reprogramming of tRNA modifications controls the oxidative stress response by codon-biased translation of proteins. Nat Commun.

[153] Preston MA, D’Silva S, Kon Y, Phizicky EM (2013). tRNAHis 5-methylcytidine levels increase in response to several growth arrest conditions in Saccharomyces cerevisiae. RNA.

[154] Endres L, Begley U, Clark R, Gu C, Dziergowska A, Małkiewicz A, Melendez JA, Dedon PC, Begley TJ (2015). Alkbh8 regulates selenocysteine-protein expression to protect against reactive oxygen species damage. PLoS One.

[155] Fernandez-Vazquez J, Vargas-Perez I, Sanso M, Buhne K, Carmona M (2013). Modification of tRNALys UUU by elongator is essential for efficient translation of stress mRNAs. PLoS Genet.

[156] Damon JR, Pincus D, Ploegh HL (2015). tRNA thiolation links translation to stress responses in *Saccharomyces cerevisiae*. Mol Biol Cell.

[157] Vendeix FA, Murphy FV, Cantara WA, Leszczyńska G, Gustilo EM, Sproat B, Malkiewicz A, Agris PF (2012). Human tRNA(Lys3)(UUU) is pre-structured by natural modifications for cognate and wobble codon binding through keto-enol tautomerism. J Mol Biol.

[158] Sierzputowska-Gracz H, Sochacka E, Malkiewicz A, Kuo K, Gehrke CW, Agris PF (1987). Chemistry and structure of modified uridines in the anticodon, wobble position of transfer RNA are determined by thiolation. J Am Chem Soc.

[159] Agris PF, Sierzputowska-Gracz H, Smith W, Malkiewicz A, Sochacka E, Nawrot B (1992). Thiolation of uridine carbon-2 restricts the motional dynamics of the transfer RNA wobble position nucleoside. J Am Chem Soc.

[160] Yamamoto Y, Yokoyama S, Miyazawa T, Watanabe K, Higuchi S (1983). NMR analyses on the molecular mechanism of the conformational rigidity of 2-thioribothymidine, a modified nucleoside in extreme thermophile tRNAs. FEBS Lett.

[161] Sundaram M, Durant PC, Davis DR (2000). Hypermodified nucleosides in the anticodon of tRNALys stabilize a canonical U-turn structure. Biochemistry.

[162] Testa SM, Disney MD, Turner DH, Kierzek R (1999). Thermodynamics of RNA-RNA duplexes with 2- or 4-thiouridines: implications for antisense design and targeting a group I intron. Biochemistry.

[163] Kumar RK, Davis DR (1997). Synthesis and studies on the effect of 2-thiouridine and 4-thiouridine on sugar conformation and RNA duplex stability. Nucleic Acids Res.

[164] Shohda K, Okamoto I, Wada T, Seio K, Sekine M (2000). Synthesis and properties of 2′-*O*-methyl-2-thiouridine and oligoribonucleotides containing 2′-*O*-methyl-2-thiouridine. Bioorg Med Chem Lett.

[165] Okamoto I, Seio K, Sekine M (2006). Incorporation of 2′-O-methyl-2-thiouridine into oligoribonucleotides induced stable A-form structure. Chem Lett.

[166] Larsen AT, Fahrenbach AC, Sheng J, Pian J, Szostak JW (2015). Thermodynamic insights into 2-thiouridine-enhanced RNA hybridization. Nucleic Acids Res.

[167] Donohue J (1969). On N–H–S hydrogen bonds. J Mol Biol.

[168] Krüger MK, Sørensen MA (1998). Aminoacylation of hypomodified tRNAGlu in vivo. J Mol Biol.

[169] Egert E, Lindner HJ, Hillen W, Boehm MC (1980). Influence of substituents at the 5 position on the structure of uridine. J Am Chem Soc.

[170] Patil A, Chan CT, Dyavaiah M, Rooney JP, Dedon PC, Begley TJ (2012). Translational infidelity-induced protein stress results from a deficiency in Trm9-catalyzed tRNA modifications. RNA Biol.

[171] Klassen R, Grunewald P, Thüring KL, Eichler C, Helm M, Schaffrath R (2015). Loss of anticodon wobble uridine modifications affects tRNA(Lys) function and protein levels in *Saccharomyces cerevisiae*. PLoS One.

[172] Patil A, Dyavaiah M, Joseph F, Rooney JP, Chan CT, Dedon PC, Begley TJ (2012). Increased tRNA modification and gene-specific codon usage regulate cell cycle progression during the DNA damage response. Cell Cycle.

[173] Bauer F, Matsuyama A, Candiracci J, Dieu M, Scheliga J, Wolf DA, Yoshida M, Hermand D (2012). Translational control of cell division by elongator. Cell Rep.

[174] Karlsborn T, Tükenmez H, Mahmud AK, Xu F, Xu H, Byström AS (2014). Elongator, a conserved complex required for wobble uridine modifications in eukaryotes. RNA Biol.

[175] Chen C, Huang B, Eliasson M, Rydén P, Byström AS (2011). Elongator complex influences telomeric gene silencing and DNA damage response by its role in wobble uridine tRNA modification. PLoS Genet.

[176] Sakai Y, Miyauchi K, Kimura S, Suzuki T (2016). Biogenesis and growth phase-dependent alteration of 5-ethoxycarbonylmethoxyuridine in tRNA anticodons. Nucleic Acids Res.

[177] Takai K (2005). Possible conformations of 5-aminomethyluridine derivatives recognizing a G at the third position of the codon. Nucleic Acids Symp Ser.

[178] Rozov A, Demeshkina N, Khusainov I, Westhof E, Yusupov M, Yusupova G (2016). Novel base-pairing interactions at the tRNA wobble position crucial for accurate reading of the genetic code. Nat Commun.

[179] Dumelin CE (2012). Discovery and biological characterization of geranylated RNA in bacteria. Nat Chem Biol.

[180] Takai K, Yokoyama S (2003). Roles of 5-substituents of tRNA wobble uridines in the recognition of purine-ending codons. Nucleic Acids Res.

[181] Hussain S, Aleksic J, Blanco S, Dietmann S, Frye M (2013). Characterizing 5-methylcytosine in the mammalian epitranscriptome. Genome Biol.

[182] Squires JE, Patel HR, Nousch M, Sibbritt T, Humphreys DT, Parker BJ, Suter CM, Preiss T (2012). Widespread occurrence of 5-methylcytosine in human coding and non-coding RNA. Nucleic Acids Res.

[183] Carlile TM, Rojas-Duran MF, Zinshteyn B, Shin H, Bartoli KM, Gilbert WV (2014). Pseudouridine profiling reveals regulated mRNA pseudouridylation in yeast and human cells. Nature.

[184] Schwartz S, Bernstein DA, Mumbach MR, Jovanovic M, Herbst RH, León-Ricardo BX, Engreitz JM, Guttman M, Satija R, Lander ES, Fink G, Regev A (2014). Transcriptome-wide mapping reveals widespread dynamic-regulated pseudouridylation of ncRNA and mRNA. Cell.

[185] Fu Y, Dominissini D, Rechavi G, He C (2014). Gene expression regulation mediated through reversible m^6^A RNA methylation. Nat Rev Genet.

[186] Lee M, Kim B, Kim VN (2014). Emerging roles of RNA modification: m(6)A and U-tail. Cell.

[187] Wang X, He C (2014). Dynamic RNA modifications in posttranscriptional regulation. Mol Cell.

[188] Delatte B, Wang F, Ngoc LV, Collignon E, Bonvin E, Deplus R, Calonne E, Hassabi B, Putmans P, Awe S, Wetzel C, Kreher J, Soin R, Creppe C, Limbach PA, Gueydan C, Kruys V, Brehm A, Minakhina S, Defrance M, Steward R, Fuks F (2016). RNA biochemistry. Transcriptome-wide distribution and function of RNA hydroxymethylcytosine. Science.

[189] Wei CM, Gershowitz A, Moss B (1976). 5′-terminal and internal methylated nucleotide sequences in Hela cell messenger RNA. Biochemistry.

[190] Dominissini D, Moshitch-Moshkovitz S, Schwartz S, Salmon-Divon M, Ungar L, Osenberg S, Cesarkas K, Jacob-Hirsch J, Amariglio N, Kupiec M, Sorek R, Rechavi G (2012). Topology of the human and mouse m6A RNA methylomes revealed by m6A-seq. Nature.

[191] Meyer KD, Saletore Y, Zumbo P, Elemento O, Mason CE, Jaffrey SR (2012). Comprehensive analysis of mRNA methylation reveals enrichment in 3′ UTRs and near stop codons. Cell.

[192] Fustin JM, Doi M, Yamaguchi Y, Hida H, Nishimura S, Yoshida M, Isagawa T, Morioka MS, Kakeya H, Manabe I, Okamura H (2013). RNA methylation-dependent RNA processing controls the speed of the circadian clock. Cell.

[193] Jia GF, Fu Y, Zhao X, Dai Q, Zheng GQ, Yang Y (2011). *N*6-Methyladenosine in nuclear RNA is a major substrate of the obesity-associated FTO. Nat Chem Biol.

[194] Zheng G, Dahl JA, Niu Y, Fedorcsak P, Huang CM, Li CJ, Vågbø CB, Shi Y, Wang WL, Song SH, Lu Z, Bosmans RP, Dai Q, Hao YJ, Yang X, Zhao WM, Tong WM, Wang XJ, Bogdan F, Furu K, Fu Y, Jia G, Zhao X, Liu J, Krokan HE, Klungland A, Yang YG, He C (2013). ALKBH5 is a mammalian RNA demethylase that impacts RNA metabolism and mouse fertility. Mol Cell.

[195] Liu JZ, Yue YN, Han DL, Wang X, Fu Y, Zhang L (2014). A METTL3-METTL14 complex mediates mammalian nuclear RNA N-6-adenosine methylation. Nat Chem Biol.

[196] Liu J, Jia G (2014). Methylation modifications in eukaryotic messenger RNA. J Genet Genomics.

[197] Wang X, Lu Z, Gomez A, Hon GC, Yue Y, Han D, Fu Y, Parisien M, Dai Q, Jia G, Ren B, Pan T, He C (2014). *N*6-methyladenosine-dependent regulation of messenger RNA stability. Nature.

[198] Liu N, Dai Q, Zheng G, He C, Parisien M, Pan T (2015). *N*(6)-methyladenosine-dependent RNA structural switches regulate RNA-protein interactions. Nature.

[199] Zhou J, Wan J, Gao X, Zhang X, Jaffrey SR, Qian SB (2015). Dynamic m(6)A mRNA methylation directs translational control of heat shock response. Nature.

[200] Meyer KD, Patil DP, Zhou J, Zinoviev A, Skabkin MA, Elemento O, Pestova TV, Qian SB, Jaffrey SR (2015). 5′ UTR m(6)A promotes cap-independent translation. Cell.

[201] Roost C, Lynch SR, Batista PJ, Qu K, Chang HY, Kool ET (2015). Structure and thermodynamics of *N*6-methyladenosine in RNA: a spring-loaded base modification. J Am Chem Soc.

[202] Wang X, Zhao BS, Roundtree IA, Lu Z, Han D, Ma H, Weng X, Chen K, Shi H, He C (2015). *N*(6)-methyladenosine modulates messenger RNA translation efficiency. Cell.

[203] Batista PJ, Molinie B, Wang J, Qu K, Zhang J, Li L, Bouley DM, Lujan E, Haddad B, Daneshvar K, Carter AC, Flynn RA, Zhou C, Lim KS, Dedon P, Wernig M, Mullen AC, Xing Y, Giallourakis CC, Chang HY (2014). m(6)A RNA modification controls cell fate transition in mammalian embryonic stem cells. Cell Stem Cell.

[204] Geula S, Moshitch-Moshkovitz S, Dominissini D, Mansour AA, Kol N, Salmon-Divon M, Hershkovitz V, Peer E, Mor N, Manor YS, Ben-Haim MS, Eyal E, Yunger S, Pinto Y, Jaitin DA, Viukov S, Rais Y, Krupalnik V, Chomsky E, Zerbib M, Maza I, Rechavi Y, Massarwa R, Hanna S, Amit I, Levanon EY, Amariglio N, Stern-Ginossar N, Novershtern N, Rechavi G, Hanna JH (2015). Stem cells. m6A mRNA methylation facilitates resolution of naïve pluripotency toward differentiation. Science.

[205] Chen T, Hao YJ, Zhang Y, Li MM, Wang M, Han W, Wu Y, Lv Y, Hao J, Wang L, Li A, Yang Y, Jin KX, Zhao X, Li Y, Ping XL, Lai WY, Wu LG, Jiang G, Wang HL, Sang L, Wang XJ, Yang YG, Zhou Q (2015). m(6)A RNA methylation is regulated by microRNAs and promotes reprogramming to pluripotency. Cell Stem Cell.

[206] Choi J, Ieong KW, Demirci H, Chen J, Petrov A, Prabhakar A, O’Leary SE, Dominissini D, Rechavi G, Soltis SM, Ehrenberg M, Puglisi JD (2016). *N*(6)-methyladenosine in mRNA disrupts tRNA selection and translation-elongation dynamics. Nat Struct Mol Biol.

[207] Hudson BH, Zaher HS (2015). *O*6-Methylguanosine leads to position-dependent effects on ribosome speed and fidelity. RNA.

[208] Dominissini D, Nachtergaele S, Moshitch-Moshkovitz S, Peer E, Kol N, Ben-Haim MS, Dai Q, Di Segni A, Salmon-Divon M, Clark WC, Zheng G, Pan T, Solomon O, Eyal E, Hershkovitz V, Han D, Doré LC, Amariglio N, Rechavi G, He C (2016). The dynamic N(1)-methyladenosine methylome in eukaryotic messenger RNA. Nature.

[209] Lovejoy AF, Riordan DP, Brown PO (2014). Transcriptome-wide mapping of pseudouridines: pseudouridine synthases modify specific mRNAs in *S. cerevisiae*. PLoS One.

[210] Zhao BS, He C (2015). Pseudouridine in a new era of RNA modifications. Cell Res.

[211] Karijolich J, Yu YT (2011). Converting nonsense codons into sense codons by targeted pseudouridylation. Nature.

[212] Roundtree IA, He C (2015). RNA epigenetics-chemical messages for posttranscriptional gene regulation. Curr Opin Chem Biol.

[213] Berulava T, Rahmann S, Rademacher K, Klein-Hitpass L, Horsthemke B (2015). *N*6-adenosine methylation in MiRNAs. PLoS One.

[214] Alarcon CR, Lee H, Goodarzi H, Halberg N, Tavazoie SF (2015). N6-methyladenosine marks primary microRNAs for processing. Nature.

[215] Grosjean H, de Crécy-Lagard V, Marck C (2010). Deciphering synonymous codons in the three domains of life: co-evolution with specific tRNA modification enzymes. FEBS Lett.

[216] O’Brien EP, Ciryam P, Vendruscolo M, Dobson CM (2014). Understanding the influence of codon translation rates on co-translational protein folding. Acc Chem Res.

[217] O’Brien EP, Vendruscolo M, Dobson CM (2014). Kinetic modelling indicates that fast-translating codons can coordinate co-translational protein folding by avoiding misfolded intermediates. Nat Commun.

[218] Yu CH, Dang Y, Zhou Z, Wu C, Zhao F, Sachs MS, Liu Y (2015). Codon usage influences the local rate of translation elongation to regulate estimated protein folding. Mol Cell.

[219] Dana A, Tuller T (2014). The effect of tRNA levels on decoding times of mRNA codons. Nucleic Acids Res.

[220] Nedialkova DD, Leidel SA (2015). Optimization of codon translation rates via tRNA modifications maintains proteome integrity. Cell.

[221] Zinshteyn B, Gilbert WV (2013). Loss of a conserved tRNA anticodon modification perturbs cellular signaling. PLoS Genet.

[222] Deng W, Babu R, Su D, Yin S, Begley TJ, Dedon PC (2015). Trm9-catalyzed tRNA modifications regulate global protein expression by codon-biased translation. PLoS Genet.

[223] Tuller T, Carmi A, Vestsigian K, Navon S, Dorfan Y, Zaborske J, Pan T, Dahan O, Furman I, Pilpel Y (2010). An evolutionarily conserved mechanism for controlling the efficiency of protein translation. Cell.

[224] López D, Pazos F (2015). Protein functional features are reflected in the patterns of mRNA translation speed. BMC Genom.

[225] Zhou M, Wang T, Fu J, Xiao G, Liu Y (2015). Nonoptimal codon usage influences protein structure in intrinsically disordered regions. Mol Microbiol.

[226] Chu D, Kazana E, Bellanger N, Singh T, Tuite MF, von der Haar T (2014). Translation elongation can control translation initiation on eukaryotic mRNAs. EMBO J.

